# Domain-Adaptive MRI Learning Model for Precision Diagnosis of CNS Tumors

**DOI:** 10.3390/biomedicines14010235

**Published:** 2026-01-21

**Authors:** Wiem Abdelbaki, Hend Alshaya, Inzamam Mashood Nasir, Sara Tehsin, Salwa Said, Wided Bouchelligua

**Affiliations:** 1 College of Engineering and Technology, American University of the Middle East, Egaila 54200, Kuwait; wiem.abdelbaki@aum.edu.kw; 2 Applied College, Imam Mohammad Ibn Saud Islamic University (IMSIU), Riyadh 11432, Saudi Arabia; hialshaya@imamu.edu.sa (H.A.); wabouchelligua@imamu.edu.sa (W.B.); 3 Human-Environment-Technology (HET) Systems Centre, Mykolas Romeris University, 08303 Vilnius, Lithuania; 4 Faculty of Informatics, Kaunas University of Technology, 51368 Kaunas, Lithuania; sara.tehsin@ktu.edu; 5 Computer Science Department, University College of Haql, University of Tabuk, Tabuk 71491, Saudi Arabia; shmida@ut.edu.sa

**Keywords:** CNS tumors, MRI, domain adaptation, brain tumor segmentation, transformer models, multicenter variability, convolutional neural network, machine learning

## Abstract

**Background:** Diagnosing CNS tumors through MRI is limited by significant variability in scanner hardware, acquisition protocols, and intensity characteristics at clinical centers, resulting in substantial domain shifts that lead to diminished reliability for automated models. **Methods:** We present a Domain-Adaptive MRI Learning Model (DA-MLM) consisting of an adversarially aligned hybrid 3D CNN–transformer encoder with contrastive regularization and covariance-based feature harmonization. Varying sequence MRI inputs (T1, T1ce, T2, and FLAIR) were inputted to multi-scale convolutional layers followed by global self-attention to effectively capture localized tumor structure and long-range spatial context, with domain adaptation that harmonizes feature distribution across datasets. **Results:** On the BraTS 2020 dataset, we found DA-MLM achieved 94.8% accuracy, 93.6% macro-F1, and 96.2% AUC, improving upon previously established benchmarks by 2–4%. DA-MLM also attained Dice score segmentation of 93.1% (WT), 91.4% (TC), and 89.5% (ET), improving upon 2–3.5% for CNN and transformer methods. On the REMBRANDT dataset, DA-MLM achieved 92.3% accuracy with segmentation improvements of 3–7% over existing U-Net and expert annotations. Robustness testing indicated 40–60% less degradation under noise, contrast shift, and motion artifacts, and synthetic shifts in scanner location showed negligible performance impairment (<0.06). Cross-domain evaluation also demonstrated 5–11% less degradation than existing methods. **Conclusions:** In summary, DA-MLM demonstrates improved accuracy, segmentation fidelity, and robustness to perturbations, as well as strong cross-domain generalization indicating the suitability for deployment in multicenter MRI applications where variation in imaging performance is unavoidable.

## 1. Introduction

Magnetic Resonance Imaging (MRI) is a crucial tool for diagnosing, grading, and planning the treatment of Central Nervous System (CNS) tumors due to its ability to acquire rich structural and anatomic information without ionizing radiation. Despite the clinical importance of MRI, the automatic analysis of CNS tumors is still an open challenge; MRI data collected at different sites typically differ considerably regarding contrast profiles, scanner hardware, magnetic field strength, coil configurations, and acquisition protocol. Such variation in distribution significantly disrupts the performance and reliability of deep learning models when deployed in practice and across multiple centers. Standard convolutional neural networks (CNNs) and transformer-based architectures continue to struggle to generalize beyond the training distribution, therefore demonstrating poor robustness at unseen domains. Such limitations have been shown in studies that consider the performance of deep diagnostic models in the presence of domain shift [1,2,3].

In the last ten years and despite the emergence of other methodologies, deep learning remained the dominant of the two paradigms for brain tumor classification and segmentation, owing to advancements in architectures (e.g., 3D U-Net variants, residual CNN architectures, and transformer architectures) and competitive performance on common benchmarks [4,5,6]. However, most state-of-the-art methods assume distributions of training data will match those used for testing, which constrains their use in classification and segmentation tasks on heterogeneous multi-institutional datasets. As multicenter research increases in size and numbers, emerging studies highlight the need for domain-resilient neural networks that can learn invariant features from disparate imaging cohorts [7,8,9]. Domain adaptation and generalization techniques—often using adversarial alignment, as well as contrastive learning, meta-learning, or statistical harmonization as objective criteria—have been identified as an effective approach for addressing differences in data characteristics; yet, many routines still find it difficult to balance the competing goals of achieving invariance while maximizing the retention of discriminative, tumor- specific information.

In addition, multi-sequence MRI presents another level of complexity. Sequences such as T1, T1ce, T2, and FLAIR can provide complementary data about tumor subregions (or features where the tumor interfaces with the brain), enhancement patterns, and edema, but the distribution of these sequences tends to vary, especially in multicenter studies. Sequence-specific normalization alone is unable to reduce the gaps in intensity ranges and noise characteristics in real datasets [10,11]. Recent research has approached this problem with attention-based fusion mechanisms, sequence-specific encoders, and hybridized CNN–transformer pipelines [12,13,14]. While these methods increase the expressiveness of features, they remain sensitive to domain drift and can have high performance variability.

Along with domain shift, robust performance to noise and artifacts is important for a safe deployment to the clinic. MRI scans often have, and are subject to, Gaussian noise, motion blur, intensity inhomogeneity, and geometric distortion. New work from recent literature demonstrates that small perturbations can seriously disrupt the predictions of standard deep models [15,16,17]. We expect models to work well under ideal conditions and to thus need some additional considerations to satisfy modern clinical model expectations of reliability and repeatability. This drives modeling exploration to create architectures that can account for perturbations and imaging artifacts in the model training.

In recent years, deep learning approaches for the MRI-based diagnosis of CNS tumors has improved with the availability of multicenter datasets and advancements in feature-learning architectures, specifically the BraTS 2020 dataset, which has become the benchmark dataset for evaluating segmentation models in various clinical imaging settings. Specific convolutional neural network (CNN) architectures including 3D U-Net and its ensemble variations have shown promising results in delineating whole tumor, tumor core, and enhancing tumor regions [18]. These models leverage encoder–decoder architectures with skip connections allowing fine anatomical detail to be preserved while learning hierarchical features. Many works have modified these architectures to add residual connections, dense blocks, and multi-sequence fusion strategies to improve generalizability across various MRI acquisition methods. Likewise, these CNN architectures have shown great results with the glioma grading task on the REMBRANDT dataset [19,20].

Even though they have been effective in capturing local texture and region-related features, CNNs have an inherent limitation with respect to modeling long-range spatial dependencies due to their limited receptive fields. As a way to overcome this shortcoming, hybrid convolution–attention architectures have emerged that utilize attention modules and vision transformers, such as channel-spatial attention, self-attention blocks, or lightweight transformer-based components that learn to increase global context awareness [21,22]. These models achieve improved segmentation consistency across heterogeneous MRI center scanning protocols, as well enhanced assessment of tumor boundary localization in infiltrative regions. Hybrid models have also demonstrated consistent performance on multicenter classification datasets like REMBRANDT by simply integrating fine-grained structural cues with global tumor growth patterns [23].

Transformer-based models have become common for CNS tumor research because of self-attention’s ability to capture temporal and long-range dependencies. In addition to ViTs, 3D transformers show significant promise in the BraTS 2020 dataset with improvements in tumor core and tumor segmentation programs [24]. Along with separating and classifying MRI image features to support classifications, transformers also enable the capture and representation of subtle morphologic differences when subtyping gliomas using REMBRANDT [25]. However, the development and training of transformers necessitated further consideration towards the challenges of patch extraction, positional embedding, and model training difficulty when supervised on heterogeneous MRI distributions. Coupled with their computationally demanding training, we propose testing additional domain-adaptive transformers pipelines.

Another important direction of research is reducing domain shift across MRI scanners and sites. Intensity normalization and statistical harmonization methods do reduce variability, but they still make accommodations for deeper structural differences. Learning-based domain adaptation methods, often utilizing adversarial learning, domain-invariant encoders, or harmonization layers, are becoming increasingly important to reduce bias caused by the scanner [26,27]. More recently, transformer-based harmonization frameworks have combined normalization and representation learning within a single architecture to jointly reduce variability and improve discriminative capabilities [28]. These learning-based frameworks consistently outperform conventional pipelines in cross-center testing, as shown in Table 1, and in addition illustrate the need for harmonization-aware MRI models.

Although deep learning has made much progress towards automatic CNS tumor diagnosis, there are many limitations still present in the research space. The conventional CNN models, while very effective at learning local tumor morphology, are less able to model long-range spatial dependencies which are necessary for understanding diffuse infiltration patterns and anatomical background. CNN-attention models do account for some of this aspect, but they also contribute to a level of model complexity that makes them difficult to optimize as an example. In recent developments, while transformer-based architectures have shown utility for establishing global representations, they largely rely on large-scale homogenous training cohorts. This tends to render some instability concerns regarding the heterogeneity, once again posing a challenge, and is problematic regarding the nature of clinical MRI datasets, which exists in limited size and heterogeneous provenance. Lastly, although there are domain adaptation approaches in deep learning, such as adversarial aligners, gradient reversal networks, and statistical normalization layers, to reduce owned scanner dispersion within a learning cohort, they come with a cost of smoothing patterns and even the clinical use of a subtle radiological characteristic, potentially even masking a clinically useful tumor phenotype. Overall, these limitations either lead to models that cannot model generalization over the variability in MRI domains or model representational fidelity potentially due to domain invariance [33].

In this study, we tackle these issues by proposing a DA-MLM, which is an integrated framework that aims to achieve both high discriminative performance and strong cross-domain generalization for CNS tumor analysis. DA-MLM is based on a hybrid 3D CNN–transformer encoder designed to capture local morphological patterns and global contextual dependencies simultaneously. In order to account for domain differences, the model uses a trilevel adaptation mechanism that includes adversarial feature alignment, instance-level contrastive regularization, and covariance-based harmonization. These complementary components jointly guide the encoder towards producing robust domain-invariant representations that are clinically meaningful. In contrast to existing methods, DA-MLM is explicitly trained and tested under intra-domain and cross-domain settings such as simulated synthetic scanner shifts and controlled perturbation experiments.

We performed an evaluation with BraTS 2020 and REMBRANDT, which are two multi-sequence MRI datasets that feature very different acquisition types. From extensive experiments, we demonstrate that DA-MLM outperforms recent state-of-the-art (SOTA) architectures over classification accuracy, segmentation performance, robustness to perturbations, and stability across domains. Contributions of this study are summarized as (1) a unified hybrid CNN–transformer backbone for multi-sequence tumor analysis, (2) a trilevel domain adaptation framework with adversarial, contrastive, and covariance-driven components, (3) a comprehensive evaluation of robustness across perturbations and scanner variations, and (4) extensive benchmark results showing superior performance and lower variance to recent model SOTA from 2023 onward.

The remainder of the paper is structured as follows: Section 2 describes the DA-MLM framework proposal, which includes the hybrid encoder, the domain alignment losses, and the joint classification–segmentation heads. Section 3 describes the extensive experiments, including in-domain and cross-domain experiments, ablation studies, robustness studies, and comparisons to state-of-the-art models. Section 4 concludes the paper with a brief summary of the findings and possible future research directions.

## 2. Proposed Methodology

**Figure 1:**
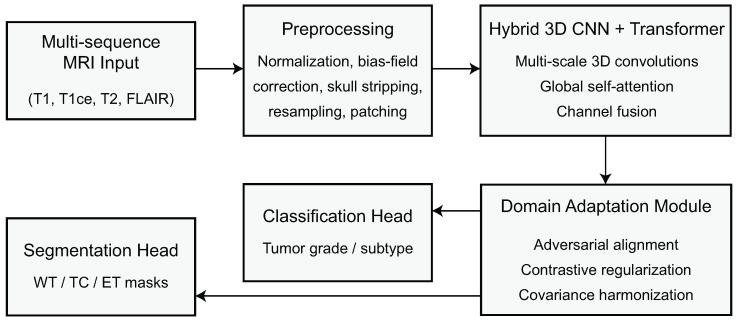
Overview of the proposed DA-MLM for CNS tumor diagnosis. The term ‘head’ refers to task-specific output branches (classification and segmentation layers) of the network and is not related to multi-head attention mechanisms.

The proposed DA-MLM aims to deliver a strong and generalizable CNS tumor diagnosis for disparate clinical domains. The model will compensate for scanner differences, inter-center intensity variation, and variations in the structural distributions of MRIs through harmonized preprocessing, adaptive feature extraction, and domain-invariant representation learning. The overall approach consists of four distinct components: MRI preprocessing, domain-aware feature extraction, adaptive representation alignment, and classification. Figure 1 presents an overview of the DA-MLM framework.

### 2.1. MRI Preprocessing Pipeline

The MRI preprocessing pipeline establishes a mathematically coherent foundation that mitigates scanner-induced variability and prepares the input volumes for subsequent domain-adaptive learning. Let an MRI volume acquired from either BraTS 2020 or REMBRANDT be denoted by $V^{\left(e\right)}\in R^{H\times W\times D}$, where e∈{T1,T1ce,T2,FLAIR} indexes the sequence type, and *H*, *W*, and *D* represent the scan dimensions in axial, coronal, and sagittal directions, respectively. To reduce intra- and inter-sequence intensity inconsistencies, each voxel $v_{i}$ within $V^{\left(e\right)}$ is normalized through z-score standardization, which transforms the voxel intensities such that the resulting distribution attains zero mean and unit variance. This normalization is expressed as

(1) $${\hat{v}}_{i}^{\left(e\right)}=\frac{v_{i}^{\left(e\right)}-\mu^{\left(e\right)}}{\sigma^{\left(e\right)}},$$

where $\mu^{\left(e\right)}$ and $\sigma^{\left(e\right)}$ denote the mean and standard deviation of the intensity distribution for the *s*-th MRI sequence. To correct low-frequency intensity inhomogeneities caused by magnetic field non-uniformities, a bias-field correction step is applied using a multiplicative model in which each observed intensity is represented as the product of a true tissue intensity and a smoothly varying bias field. This can be written as

(2) $$v_{i}^{\left(e\right)}=b_{i}^{\left(e\right)}\cdot t_{i}^{\left(e\right)}+\epsilon_{i}^{\left(e\right)},$$

where $b_{i}^{\left(e\right)}$ is the estimated bias-field component, $t_{i}^{\left(e\right)}$ is the underlying true signal to be recovered, and $\epsilon_{i}^{\left(e\right)}$ captures high-frequency noise. After skull stripping, which removes non-brain voxels from $V^{\left(e\right)}$ through a binary brain mask $M\in {\left\{0,1\right\}}^{H\times W\times D}$, the valid brain region is defined as $V_{brain}^{\left(e\right)}=V^{\left(e\right)}⊙M$, where ⊙ denotes element-wise multiplication. A spatial resampling operator R(·) is then applied to achieve isotropic voxel spacing; this ensures consistent geometric representations across datasets and is expressed as

(3) $${\tilde{V}}^{\left(e\right)}=R\left(V_{brain}^{\left(e\right)},\delta \right),$$

where δ denotes the target isotropic resolution. To align all volumes to a common anatomical reference space, a spatial registration function T(·) is applied such that

(4) $${\bar{V}}^{\left(e\right)}=T\left({\tilde{V}}^{\left(e\right)},A\right),$$

where *A* is the affine or deformable transformation that maps the MRI volume into the chosen template space. Subsequently, all four sequences are concatenated channel-wise to form a unified multi-sequence tensor $X\in R^{H^{\prime }\times W^{\prime }\times D^{\prime }\times 4}$, defined as

(5) $$X=\left[\;{\bar{V}}^{(T1}\right),\;{\bar{V}}^{\left(T1ce\right)},\;{\bar{V}}^{(T2}),\;{\bar{V}}^{(FLAIR})\;].$$

**Figure 2:**
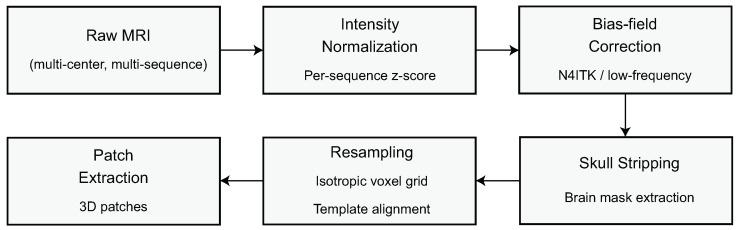
MRI preprocessing pipeline used for BraTS 2020 and REMBRANDT.

To enable effective training, while also preserving anatomical continuity, volumetric patches are collected using a sliding window operator P(·) which samples overlapping volume size h×w×d compositions on the spatial grid. This extraction is graphically demonstrated as

(6) $$X_{k}=P\left(X,k;h,w,d,\tau \right),$$

where *k* identifies the location of the patches and the variable τ denotes the stride that determines the amount of overlap between patches. Each variable introduced in this preprocessing phase has a specific purpose: $X_{k}$ is the base input to the domain-aware feature extraction backbone, $\left(\mu^{\left(e\right)},\sigma^{\left(e\right)}\right)$ are the normalization parameters to standardize voxel intensities acquired at different sites, $b_{i}^{\left(e\right)}$ refer to the bias-field term to promote scanner stability, and *A* reflects the registration transformation to render anatomical comparability across subjects. The preprocessing steps work collectively to create a mathematically consistent domain-harmonized representation of the content internally that would subsequently support the domain-invariant learning processes described in the subsequent subsections. The overall preprocessing sequence is depicted in Figure 2.

### 2.2. Domain-Aware Feature Extraction Backbone

The domain-aware feature extraction backbone is designed to transform the preprocessed MRI patches $X_{k}\in R^{h\times w\times d\times 4}$ into a compact yet discriminative latent representation that captures both local anatomical structure and global contextual dependencies across brain regions. The backbone integrates a 3D convolutional stem for local feature extraction with transformer-based attention layers for long-range dependency modeling. Let the convolutional stem be denoted as a function C(·) composed of $L_{c}$ multi-scale residual blocks, each operating on distinct spatial receptive fields to ensure that fine-grained tumor morphology and broad anatomical cues are simultaneously preserved. The output of the convolutional stem is a feature tensor $F_{0}\in R^{h^{\prime }\times w^{\prime }\times d^{\prime }\times C}$ defined as

(7) $$F_{0}=C\left(X_{k}\right)=R_{L_{c}}\left(R_{L_{c}-1}\left(\ldots R_{1}\left(X_{k}\right)\ldots \right)\right),$$

where $R_{\ell }$ denotes the *ℓ*-th residual block and *C* is the channel dimensionality produced by the final convolutional layer. Each residual block follows the formulation

(8) $$R_{\ell }\left(U\right)=U+\phi_{\ell }\left(W_{\ell }*U\right),$$

in which $W_{\ell }$ is a 3D convolution kernel, * denotes convolution, and $\phi_{\ell }\left(\cdot \right)$ is a nonlinear activation function, typically a parametric rectified linear unit. This design guarantees gradient stability, diminishes vanishing gradient issues, and increases the network’s capacity for multi-scale tumor feature extraction. To exploit global contextual information, the tensor $F_{0}$ is reshaped into a sequence of spatial tokens for transformer decoding. Let $Z_{0}\in R^{N\times C}$ denote the sequence of tokens where $N=h^{\prime }w^{\prime }d^{\prime }$ is the number of spatial positions. Positional encodings $P\in R^{N\times C}$ are added to preserve volumetric spatial structure, thus giving

(9) $$Z_{0}=reshape\left(F_{0}\right)+P.$$

The transformer encoder consists of $L_{t}$ stacked self-attention blocks. At each layer *l*, the input $Z_{l-1}$ is projected into query, key, and value matrices using learned projections $W_{l}^{Q}$, $W_{l}^{K}$, and $W_{l}^{V}$:

(10) $$Q_{l}=Z_{l-1}W_{l}^{Q},\;K_{l}=Z_{l-1}W_{l}^{K},\;V_{l}=Z_{l-1}W_{l}^{V}.$$

Self-attention computes similarity scores across all token pairs, enabling the model to learn spatial dependencies that transcend local convolutional receptive fields. The attention operator is formulated as

(11) $$Attn\left(Q_{l},K_{l},V_{l}\right)=softmax\left(\frac{Q_{l}K_{l}^{⊤}}{\sqrt{C}}\right)V_{l}.$$

The output of the *l*-th transformer layer is then computed using a residual connection followed by a feed-forward network $F_{l}\left(\cdot \right)$:

(12) $$Z_{l}=Z_{l-1}+F_{l}\left(Attn(Q_{l},K_{l},V_{l})\right).$$

**Figure 3:**
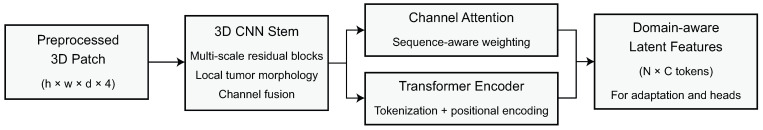
Hybrid 3D CNN–transformer feature extraction backbone. Preprocessed 3D patches are first processed by a 3D CNN stem with multi-scale residual blocks and channel fusion. The resulting features are fed to a channel attention module and transformer encoder, producing domain-aware latent tokens that capture both local tumor morphology and global anatomical context.

To fuse complementary information from the four MRI sequences, a channel attention mechanism is applied to the convolutional stem output before tokenization. Let $s\in R^{C}$ denote a learned channel importance vector computed as

(13) $$s=\sigma \left(W_{2}\;\delta \left(W_{1}\;GAP\left(F_{0}\right)\right)\right),$$

where GAP(·) is global average pooling, δ(·) is a ReLU activation, σ(·) is a sigmoid function, and $W_{1},W_{2}$ are trainable matrices. The channel-refined feature tensor is then given by

(14) $$F_{0}^{*}\left(x,y,z,c\right)=s_{c}\cdot F_{0}\left(x,y,z,c\right),$$

making sure the backbone responsible for feature extraction can selectively amplify informative channels associated with subtle tumor-enhancing regions and edema patterns. $s=[s_{c}]$ denotes the learned channel-importance vector produced by the channel attention module, where $s_{c}\in \left(0,1\right)$ represents the sigmoid-normalized weighting coefficient applied to the *c*-th feature channel. The combination of convolutional and transformer architecture generates a domain-aware latent encoding that captures multi-scale structural patterns, strengthens inter-sequence complementarities, and leverages global spatial context gleaned from the volumetric MRI input. This encoded representation forms the basis for the domain-invariant learning strategy described in the following subsection, where the extracted features are aligned across clinical domains to ensure robust and stable CNS tumor diagnosis. The architecture of the hybrid CNN–transformer encoder is illustrated in Figure 3.

### 2.3. Adaptive Domain-Invariant Representation Learning

The adaptive domain-invariant representation learning system aims to guarantee that the latent features extracted from the hybrid convolution–transformer encoder remain consistent and transferable across heterogeneous MRI domains (e.g., BraTS 2020 and REMBRANDT). Let us denote the encoded representation of an input patch $X_{k}$ by $Z_{L_{t}}\in R^{N\times C}$, where *N* is the number of spatial tokens, and *C* is the feature dimensionality resulting from the last transformer layer. The principal aim of this module is to guarantee that these representations still embody tumor-related information while discarding domain-specific style information due to scanner hardware, acquisition protocols, and postprocessing differences. To achieve this, the framework adopts an adversarial domain learning mechanism coupled with an instance-based contrastive regularization strategy, which both push the encoder towards learning domain-agnostic features.

The adversarial component introduces a domain discriminator D(·), which distinguishes which dataset each encoded representation belongs to. We denote the domain label as $y_{d}\in \left\{0,1\right\}$, where 0 corresponds to BraTS 2020 and 1 corresponds to REMBRANDT. The discriminator takes the concatenated representation $g=GAP(Z_{L_{t}})$, where GAP(cdot) denotes global average pooling of all tokens, and predicts the domain probability ${\hat{y}}_{d}=D\left(g\right)$. The discriminator is trained using a binary cross-entropy loss function.

(15) $$L_{disc}=-\left[\;y_{d}\;\log\left({\hat{y}}_{d}\right)+\left(1-y_{d}\right)\;\log\left(1-{\hat{y}}_{d}\right)\;\right],$$

**Figure 4:**
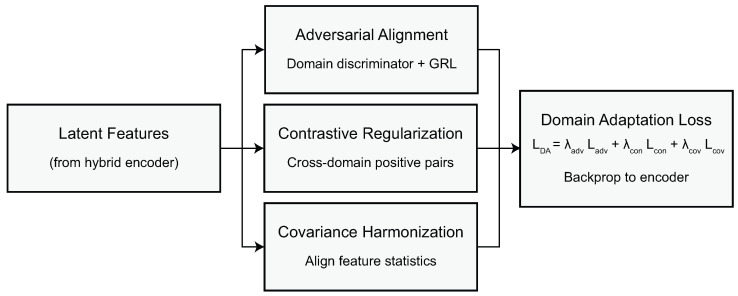
Domain adaptation module operating on latent features from the hybrid encoder. The module combines adversarial alignment via a domain discriminator and gradient reversal layer, contrastive regularization over cross-domain positive pairs, and covariance harmonization to align feature statistics, which are aggregated into a single domain adaptation loss used to update the encoder.

Here, ${\hat{y}}_{d}$ indicates the class prediction from the discriminator regarding the domain. Meanwhile, the encoder is trying to create features that confuse the discriminator (indicate uncertainty) updated via the adversarial feature alignment loss.

(16) $$L_{adv}=-\left[\;\frac{1}{2}\log\left({\hat{y}}_{d}\right)+\frac{1}{2}\log\left(1-{\hat{y}}_{d}\right)\;\right],$$

which incentivizes ${\hat{y}}_{d}$ to become close to $\frac{1}{2}$ so that domain indistinguishability is established in the latent space. The gradient reversal rule guarantees that the method constructs a two-player minimax game, with the discriminator minimizing $L_{disc}$ while the encoder minimizes $L_{adv}$, in the interest of aligning the statistical properties of the features of both domains. In addition to adversarial alignment, we use a contrastive regularization framework at the instance level to make sure that anatomically similar samples from different domains may encode to more proximal representations while reasonably distant from anatomically dissimilar examples. Let $z_{i}$ and $z_{j}$ be normalized feature representations extracted from two patches representing similar anatomical properties from distinct domains (i.e., $z_{i}\in O$ where the patch is from the source domain and $z_{j}\in U$ where the patch is from the target domain).

(17) $$s_{ij}=\frac{z_{i}^{⊤}z_{j}}{\tau },$$

where τ>0 is a temperature parameter that regulates the smoothness of the similarity distribution. For each anchor feature $z_{i}$, there is a positive sample $z_{i}^{+}$ from the alternate domain, which has the same anatomical/intensity structure, while the negative samples $z_{i}^{-}$ come from independent, unrelated patches. The contrastive loss that governs this alignment can be specified as

(18) $$L_{con}=-\log\frac{\exp(s_{ii^{+}})}{\exp\left(s_{ii^{+}}\right)+\sum_{n}\exp\left(s_{in}\right)},$$

where the denominator sums similarity scores across all negative samples. Here, $s_{ij}$ indicates the similarity score between feature embeddings $z_{i}$ and $z_{j}$. The notation $s_{ii}^{+}$ refers to the similarity score of a positive cross-domain pair, i.e., two anatomically corresponding samples drawn from different domains. Conversely, $s_{in}$ represents the similarity score between the anchor patch $z_{i}$ and a negative sample $z_{n}$ that is anatomically unrelated. The standalone symbol *s* used in the attention module (Equation (13)) is unrelated to the scalar similarity scores $s_{ij}$ used in the contrastive formulation; the former denotes a vector of channel weights, whereas the latter represents pairwise cosine similarities in embedding space. This loss term promotes invariance to domain shifts while maintaining discriminating power for downstream tumor classification. A covariance alignment term is added to further regularize training, such that the second-order feature statistics are aligned across domains. Let $\Sigma_{B}$ and $\Sigma_{R}$ denote the covariance matrices of the latent features for BraTS and REMBRANDT. The alignment is enforced by utilizing the following equation:

(19) $$L_{cov}={∥\Sigma_{B}-\Sigma_{R}∥}_{F}^{2},$$

where ${∥\cdot ∥}_{F}$ stands for the Frobenius norm. This loss guarantees that the geometric structure of latent space is preserved across datasets. Putting together these components, the total domain-invariant learning objective is defined as

(20) $$L_{DA}=\lambda_{adv}L_{adv}+\lambda_{con}L_{con}+\lambda_{cov}L_{cov},$$

where $\lambda_{adv}$, $\lambda_{con}$, and $\lambda_{cov}$ signify weighting hyperparameters to balance how much adversarial alignment, contrastive regularization, and covariance matching influence the overall loss. This composite loss function guarantees that the encoder can learn a unified, domain-agnostic representation, so that the diagnostic classification module that follows can effectively operate, no matter what dataset it originates from. Through this process, the model should then learn tumor-relevant features that are invariant across multicenter MRI, which is a strong basis for high-fidelity CNS tumor diagnosis. The multilevel domain adaptation mechanism is detailed in Figure 4.

### 2.4. Diagnostic Classification Layer

The diagnostic classification layer constitutes the final predictive stage of the proposed domain-adaptive framework, transforming the domain-aligned latent representation into clinically relevant tumor labels. Let $Z_{L_{t}}\in R^{N\times C}$ denote the output of the adaptive domain-invariant encoder, where *N* is the number of spatial tokens and *C* the embedding dimension. To reduce spatial redundancy while preserving global contextual information, a global average pooling operator GAP(·) aggregates these tokens into a compact feature vector $h\in R^{C}$ defined as

(21) $$h=GAP\left(Z_{L_{t}}\right)=\frac{1}{N}\sum_{i=1}^{N}z_{i},$$

where $z_{i}$ denotes the *i*-th token. This aggregation ensures that the overall representation contains an amalgamated representation of morphological and structural patterns while being robust to small spatial changes or discrepancies across datasets. The vector *h* is then passed through a fully connected transformation $F_{\theta }\left(\cdot \right)$ parameterized by weight matrix $W_{c}\in R^{C\times K}$ and bias $b_{c}\in R^{K}$, where *K* is the number of diagnostic classes, producing the class logits $o\in R^{K}$:

(22) $$o=F_{\theta }\left(h\right)=W_{c}^{⊤}h+b_{c}.$$

**Figure 5:**
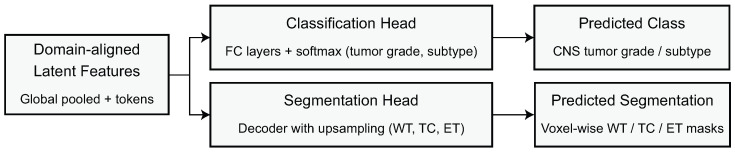
Dual diagnostic heads attached to the domain-aligned latent features. The classification head consists of fully connected layers with softmax for CNS tumor grade or subtype prediction, while the segmentation head is a decoder with upsampling layers that produces voxel-wise WT, TC, and ET masks.

The logits are converted into predictive probabilities through a softmax mapping, yielding the class probability distribution $\hat{y}=\left({\hat{y}}_{1},\ldots ,{\hat{y}}_{K}\right)$ defined as

(23) $${\hat{y}}_{k}=\frac{\exp(o_{k})}{\sum_{j=1}^{K}\exp\left(o_{j}\right)},$$

where ${\hat{y}}_{k}$ denotes the predicted probability of class *k*. For BraTS 2020, these classes correspond either to segmentation-derived tumor grades or to binary high-grade versus low-grade glioma labels depending on the experimental protocol. For REMBRANDT, the same formulation supports multi-class tumor classification or glioma subtype prediction, including astrocytoma, oligodendroglioma, and mixed glioma categories. The classification module is trained using a categorical cross-entropy objective that penalizes deviations between predicted probabilities and ground-truth labels. Let $y\in {\left\{0,1\right\}}^{K}$ represent the one-hot encoded ground-truth label. The supervised loss is expressed as

(24) $$L_{cls}=-\sum_{k=1}^{K}y_{k}\log\left({\hat{y}}_{k}\right),$$

which encourages the predicted distribution to concentrate mass on the correct diagnostic category. This supervised objective is optimized jointly with the domain adaptation loss introduced in the previous subsection, such that the encoder must simultaneously learn tumor-discriminative and domain-invariant feature representations. Let $L_{DA}$ denote the aggregated domain alignment loss. The total learning objective for the diagnostic stage is formulated as

(25) $$L_{total}=L_{cls}+\lambda_{DA}L_{DA},$$

where $\lambda_{DA}$ is a balancing factor that determines the trade-off between accuracy in prediction and robustness to cross-domain features. In order to facilitate stability and efficient convergence of the model, we optimize the model parameters using the AdamW optimizer which decouples the weight decay from the updates of the gradient and prevents over-regularization of important high-level feature dimensions. The learning rate is optimized using a cosine decay schedule as follows:

(26) $$\eta \left(t\right)=\eta_{0}\cdot \frac{1}{2}\left(1+\cos\left(\frac{\pi t}{T}\right)\right),$$

where $\eta_{0}$ is the starting learning rate, *t* is the number of training iterations, and *T* is the total number of iterations. Cosine decay allows for a gradual way of annealing the learning rate and minimizes oscillatory effects in the late stages of optimization. Balanced minibatch construction is needed to help preserve domain invariance. For each batch, MRI patches from both BraTS 2020 and REMBRANDT are sampled, such that the encoder always receives mixed-domain input, thereby enhancing adversarial and contrastive alignment constraints. Formally, let $B_{B}$ and $B_{R}$ denote random samples from one of the datasets and then each batch is constructed as

(27) $$B=B_{B}\cup B_{R},$$

ensuring that domain alignment gradients propagate consistently throughout training. By jointly optimizing over mixed-domain batches, the classifier learns to rely on domain-agnostic features rather than dataset-specific artifacts, ultimately yielding a robust, stable, and clinically transferable diagnostic model applicable to diverse MRI acquisition environments. The shared latent representation feeds into separate classification and segmentation heads as shown in Figure 5.

### 2.5. Training Protocol and Optimization

The training protocol is designed to jointly optimize the supervised diagnostic objective and the domain adaptation objectives while ensuring numerical stability, generalization, and robustness across heterogeneous MRI sources. Let Θ denote the full set of trainable parameters across the convolutional stem, transformer layers, domain discriminator, and classification head. Training proceeds end-to-end, meaning that gradients propagate from the diagnostic classification layer through the domain-aligned representation space and back into the feature extraction backbone. This overall optimization makes it possible for the model to learn to obtain feature embeddings that not only capture tumor-discriminative features but also are invariant to differences in acquisition that are dataset-specific. Considering the total loss function $L_{total}$, which then contains both supervised classification and domain adaptation terms, we aim to train the model to minimize the following global objective:

(28) $$\Theta^{*}=\arg\min_{\Theta }\;L_{total}=\arg\min_{\Theta }\left(L_{cls}+\lambda_{DA}\;L_{DA}\right),$$

where $\lambda_{DA}$ regulates the strength of the balance between diagnostic relevance and domain invariance. To avoid overfitting to either BraTS 2020 or REMBRANDT separately, the training paradigm uses mixed domain minibatches so that each forward pass goes through a heterogeneous set of MRI distributions for the encoder and discriminator. We denote the minibatch at iteration *t* as $B^{\left(t\right)}$ and assume the samples of this minibatch are drawn from both MRI distributions, reflecting the cross-domain behavior of the gradients. Formally, for BraTS samples $B_{B}^{\left(t\right)}$ and REMBRANDT samples $B_{R}^{\left(t\right)}$, the minibatch is defined as

(29) $$B^{\left(t\right)}=B_{B}^{\left(t\right)}\cup B_{R}^{\left(t\right)},$$

and the model calculates joint gradients from this composite distribution. This strategy keeps the optimizer on-track and away from dataset-specific minima while promoting a consistent feature alignment as training proceeds. To further facilitate generalization, structured data augmentation is applied to each input patch. Let A(·) denote the augmentation operator consisting of elastic deformations that are randomized, intensity perturbations, affine rotations, and patch dropout, and let the augmented patch be given as

(30) $${\tilde{X}}_{k}=A\left(X_{k}\right),$$

where $X_{k}$ refers to the original patch after being downsampled. Elastic deformations simulate realistic anatomical variance while random rotations and intensity shifts help mitigate the possibility of the model becoming overly reliant on one specific scanner orientation and contrast setup. Patch dropout is performed by randomly masking sub-regions in the patch to augment robustness to missing or corrupted structure information. Altogether, these augmentations help broaden the sample distribution and provide support against overfitting to the tiny, little, and sometimes meaningless irregularities in the dataset. The optimization is performed using the AdamW optimizer, a first-order gradient descent algorithm, that efficiently decouples L2 weight decay from the adaptive moment estimation, allowing for stable updates to the parameters of the CNN and transformer. Define η(t) as the learning rate for the t-th iteration using a cosine decay schedule:

(31) $$\eta \left(t\right)=\eta_{min}+\frac{1}{2}\left(\eta_{0}-\eta_{min}\right)\left(1+\cos\left(\frac{\pi t}{T}\right)\right),$$

where $\eta_{0}$ is the initial learning rate, $\eta_{min}$ is the lower threshold, and *T* is the number of overall training iterations. This schedule decreases the learning rate gradually, without ejecting the learning beyond the first part of training, and allowing for smooth convergence later. Candidates are updated according to the AdamW rule:

(32) $$\Theta_{t+1}=\Theta_{t}-\eta \left(t\right)\cdot \frac{{\hat{m}}_{t}}{\sqrt{{\hat{v}}_{t}}+\epsilon }-\eta \left(t\right)\cdot \lambda_{w}\Theta_{t},$$

where ${\hat{m}}_{t}$ and ${\hat{v}}_{t}$ are the bias-corrected first and second moment estimates, $\lambda_{w}$ is the weight decay factor, and ϵ is a small constant for numerical stability. This formulation jointly controls both gradient-driven refinement and regularization. The batch normalization layers within the convolutional stem leverage domain-agnostic statistics to prevent dataset-specific drifts in activation. Let $\mu_{bn}$ and $\sigma_{bn}$ denote the batch mean and variance, respectively; rather than computing these values independently per-domain, they are estimated jointly across $B^{\left(t\right)}$:

(33) $$\mu_{bn}=\frac{1}{|B^{\left(t\right)}|}\sum_{i\in B^{\left(t\right)}}f_{i},\;\sigma_{bn}^{2}=\frac{1}{|B^{\left(t\right)}|}\sum_{i\in B^{\left(t\right)}}{\left(f_{i}-\mu_{bn}\right)}^{2},$$

where $f_{i}$ are the activations of the intermediate layers. We intentionally avoid encoding statistical signatures specific to the dataset, which ensures that we learn domain-invariant representations. We apply early stopping to avoid overfitting to the validation dataset and support stable convergence of the network, and for this we monitor a combined validation metric for both of the datasets. Let $\alpha_{B}$ and $\alpha_{R}$ be the accuracies on the validation BraTS 2020 and REMBRANDT datasets, respectively. The unified validation score used for monitoring is

(34) $$\alpha_{val}=\gamma \alpha_{B}+\left(1-\gamma \right)\alpha_{R},$$

where γ controls the relative weighting. Training terminates when $\alpha_{val}$ fails to improve for a predetermined number of epochs, ensuring that the final model selected is robust across both domains and not over-optimized for one dataset at the expense of the other. This training protocol integrates supervised learning, adversarial domain alignment, contrastive regularization, and careful optimization strategies into a cohesive end-to-end procedure that produces a domain-adaptive MRI classifier capable of maintaining stable performance across diverse clinical environments. The joint optimization of classification, segmentation, and adaptation losses is depicted in Figure 6.

## 3. Results

The results section will provide an extensive assessment of the proposed DA-MLM on both the BraTS 2020 and REMBRANDT datasets. The assessment will report around five further prepared main areas: (1) dataset characterization and evaluation protocol, (2) baseline comparison with recent state-of-the-art methods, (3) cross-domain assessment of generalization performance, (4) ablation studies to quantify the theoretical contribution of each component of the proposed methodology, and (5) robustness analysis against perturbations and domain shift. Each subsection will emphasize the contribution of the proposed domain-adaptive methodology by illustrating improvements in diagnostic stability, consistency, and what prediction can be made based on distributed clinical imaging variability.

### 3.1. Experimental Setup and Evaluation Metrics

The purpose of the experimental protocol is to carefully and systematically test the performance, robustness, and generalizability of the proposed DA-MLM across two distinct datasets, BraTS 2020 and REMBRANDT. The two datasets differ considerably with respect to acquisition protocols, anatomical coverage, subject-level class distributions, and scanner characteristics; therefore, it is important to create and use training, validation, and testing partitions that are balanced and reproducible. In the case of BraTS 2020, the preprocessed multi-sequence MRI volumes are split into training, validation, and testing subsets, in a stratified (70%,15%,15%) manner, to preserve the proportions of low-grade and high-grade glioma samples. The REMBRANDT dataset will be partitioned similarly on an 80%,10%,10% split, in consideration of its smaller sample size and heterogeneous subtype distributions. Let $D_{B}^{train},D_{B}^{val},D_{B}^{test}$ denote the BraTS training, validation, and test sets, respectively, and similarly let $D_{R}^{train},D_{R}^{val},D_{R}^{test}$ represent the corresponding REMBRANDT sets. Formally, the splits satisfy

(35) $$|D_{B}^{train}\left|:\right|D_{B}^{val}\left|:\right|D_{B}^{test}|=0.70:0.15:0.15,$$

(36) $$|D_{R}^{train}\left|:\right|D_{R}^{val}\left|:\right|D_{R}^{test}|=0.80:0.10:0.10,$$

to ensure reproducibility and consistent sampling across experimental trials. During training, mixed-domain minibatches are sampled from $D_{B}^{train}$ and $D_{R}^{train}$ uniformly to equalize the contributions of each domain dataset. Let $B_{t}$ denote the minibatch sampled at iteration *t*; then, every minibatch satisfies

(37) $$B_{t}=\left\{X_{i}∼D_{B}^{train}\right\}\cup \left\{X_{j}∼D_{R}^{train}\right\},$$

with an equal probability of obtaining samples from each of the domains in order to encourage alignment between the two domains. There are stark differences between the distributions of each of the datasets. The BraTS 2020 dataset consists of multi-sequence T1, T1ce, T2, and FLAIR volumes, which have a voxel spacing of typically between 1.0 and 1.5 mm, and the REMBRANDT dataset consists of T1- and T2-weighted clinical MRIs, which have more variability in resolution and anisotropic spacing. Let the distributions of each dataset be denoted by probability measure $p_{B}\left(X\right)$ and $p_{R}\left(X\right)$. The goal of the domain-aware backbone is to reduce the distance between these distributions at the feature level, which motivates consideration of cross-domain generalization alongside in-domain performance. Evaluation metrics will be defined to assess both multi-class diagnostic performance and robustness across heterogeneous domains. Let ${\hat{y}}_{i}$ and $y_{i}$ be the predicted and true labels, respectively, of sample *i*. Overall accuracy is given by

(38) $$Acc=\frac{1}{N}\sum_{i=1}^{N}I\left({\hat{y}}_{i}=y_{i}\right),$$

Here, *N* is used to indicate the total number of test samples; I(·) indicates indicator function. To address class imbalance (which is relevant to REMBRANDT, as some subtypes may have more cases than others), we used the macro-averaged F1-score:

(39) $$F1_{macro}=\frac{1}{K}\sum_{k=1}^{K}\frac{2\;Precision_{k}\;Recall_{k}}{Precision_{k}+Recall_{k}},$$

where *K* is the number of classes. The ROC–AUC metric is defined for each class by measuring the area under the class-wise receiver operating characteristic curve:

(40) $$AUC_{k}=\int_{0}^{1}TPR_{k}\left(\alpha \right)\;dFPR_{k}\left(\alpha \right),$$

with $TPR_{k}$ and $FPR_{k}$ denoting true positive and false positive rate functions parameterized by decision threshold α. Sensitivity and specificity are computed as

(41) $$Sensitivity=\frac{TP}{TP+FN},\;Specificity=\frac{TN}{TN+FP},$$

where TP, TN, FP, and FN denote true positive, true negative, false positive, and false negative, respectively. To quantify domain robustness, balanced accuracy is also reported:

(42) $$BalAcc=\frac{1}{2}\left(Sensitivity+Specificity\right),$$

indicating performance stability across class distributions. All experiments are performed on an NVIDIA RTX 5070 GPU with 12 GB of RAM, and the pre-training model was trained with a batch size of 16, using a cosine-decayed learning rate schedule (maximum 200 epochs). The weight was initialized with Kaiming uniform initialization. The random seed was fixed, and all experiments were repeated five times for each experimental condition, with the mean ± standard deviation of all metrics being reported. This thorough experiment design allows for a reliable evaluation to benchmark performance of the proposed domain-adaptive model, allows for fair comparison of the results to the previous state-of-the-art methods, and provides this to demonstrate robust evaluation across two clinically divergent MRI datasets. Computational efficiency of DA-MLM was quantified on an NVIDIA RTX 5070 (12 GB). The complete network requires approximately 82 GFLOPs per forward pass for a standard multi-sequence 3D volume. Average inference latency was ≈85 ms per volume (11–12 volumes/s), while end-to-end training for 200 epochs required ≈9.2 h per dataset with peak GPU memory consumption of 9.6 GB. Table 2 presents the complete hyperparameter configuration used for DA-MLM training.

In addition to reporting the mean ± standard deviation over five independent runs, paired two-tailed t-tests were conducted between DA-MLM and each baseline method for all primary evaluation metrics (classification accuracy, macro-F1, AUC, and Dice scores). Differences were considered statistically significant at p<0.05.

### 3.2. Performance on BraTS 2020 and REMBRANDT

To evaluate the proposed domain-adaptive model, thorough analyses were performed on BraTS 2020 and REMBRANDT to investigate intra-domain diagnostic consistency, segmentation performance, and statistical performance stability across imaging environments. The analyses provided in this section evaluate model performance within each dataset, as opposed to cross-domain inquiry which typically quantifies transfer robustness. In that context, the empirical performance assessment could indicate the extent to which hybrid backbone and aligned-based representation learning causes inconsistency when domain invariance is less of an issue. The model was trained and tested separately on each dataset, utilizing the standardized training–validation–testing splits described above. There were five independent runs for each experimental configuration. The following performance analyses confirm that empirical performance reflects the intended predictive outcomes, as well as the possible variability from the stochasticity of optimization and data sampling, and augmentation

The dense classification results that are detailed in Table 3 provide a high-level overview of the relative contribution of each component related to the architectural and adaptation aspects to overall diagnostic performance on BraTS 2020. Overall, the DA-MLM model performed the best on measures of overall accuracy, macro-F1 score, and balanced accuracy, indicating that the effect of hybrid feature extraction and multilevel adaptation contributed to the most consistent and separable representations. When the architecture was modified to only a CNN backbone, performance declined considerably on all metrics, suggesting that the capability for global modeling provided by the transformer encoder had some importance. The performance of the model using only a transformer backbone still outperformed the CNN baseline model albeit not the original full model. This suggests that convolutional layers may capture local morphological cues that are still informative for tumor characterization. Performance remained high when individual domain adaptation components were removed from the model, given that each component precipitates varying declines of performance: antagonistic alignment reduces the degree of consistency across domain adaptation, removing contrastive regularization diminishes instance-level feature similarity, and removing covariance matching bursts the equilibrium of the second-order statistics used for alignment. The greatest degree of relative performance decline among the domain adaptation components is observed when covariance matching was removed, introducing its significance in harmonizing the feature distributions from heterogeneous scanners.

The segmentation results in Table 4 reinforce the complementary contributions of both architectural design and domain adaptation via the proposed DA-MLM framework. The full model produces the highest Dice and Jaccard scores in every tumor subregion—whole tumor (WT), tumor core (TC), and enhancing tumor (ET)—indicating the model has a strong capability of downloading fine morphological boundaries while also maintaining coherent, global context. When the model is reduced to a CNN-only backbone, segmentation reports notably low accuracy, which highlights the restricted capability of only convolutionally derived sequences to model complex spatial dependencies (e.g., in regions with diffuse or irregular tumor infiltration). The transformer-only backbone performs better than the CNN baseline, particularly in WT and TC, reinforcing the value of long-range attention mechanisms; however, lower ET performance relative to WT indicates challenges encoding subtle, high-frequency enhancements of the tumor when disentangled from local CNN derived signals.

Classification results for the REMBRANDT dataset presented in Table 5 exhibit sharper consistency with the findings from BraTS 2020 while revealing greater complexity in terms of the more heterogeneous characteristics of REMBRANDT MRI. The DA-MLM architecture produces superior performance metrics across all compliance evaluation metrics (e.g., accuracy, macro-F1, AUC, and balanced accuracy) because it explicitly accounts for the subtleties in subtype distinctions and the unequal imaging conditions observed for the experiments. The implementation of the CNN-only backbone architecture decreases and falls substantially along all compliance metrics, indicating that convolutional features exclusively are inherently insufficient in accounting for the broader anatomical context necessary for reliable subtype classifications that require additional inter-sequence relationships in imaging. While the transformer-only option produces moderate improvements over the CNN-only condition, it still performs substantially worse than the additional imaging context and meaningful inter-sequence relationships offered by the DA-MLM design. Nevertheless, convolutional components appear critically related to the anatomical definition needed to represent locally, as the CNN backbone provides necessary edge characteristics for distinguishing more subtle tumor characteristics.

The segmentation results of the REMBRANDT dataset reported in Table 6 demonstrate the pronounced performance advantages of the full DA-MLM framework over each ablated configuration. Not only does the full DA-MLM model report the greatest Dice and Jaccard coefficients in tumor, core, and necrotic radiological features, it also appears to capture both the global tumor morphology, along with the subtle and meaningful structural differences that are inherent in heterogeneous MRI radiological images. The performance does significantly drop with either the CNN or transformer backbones used alone, indicating that localization of spatial sensitivity and modeling of global context are critical for adequate delineation of a complex tumor subregion. Removing components of domain adaptation further accentuated drops in performance for each metric, with necrosis segmentation being the most impacted. This likely reflects the sensitivity of the REMBRANDT dataset’s inherently high variability in imaging characteristics towards inconsistencies across domains. The `Without Covariance Matching’ configuration reported the largest reduction in performance metrics across all three MRI features, indicating the importance of modeling and aligning second-order statistics in order to produce stable segmentation across imaging centers. Overall, these analyses support that the successful integration of hybrid feature extraction and trilevel domain adaptation are necessary to achieve accurate and robust volumetric segmentation across a highly heterogeneous REMBRANDT dataset.

**Figure 7:**
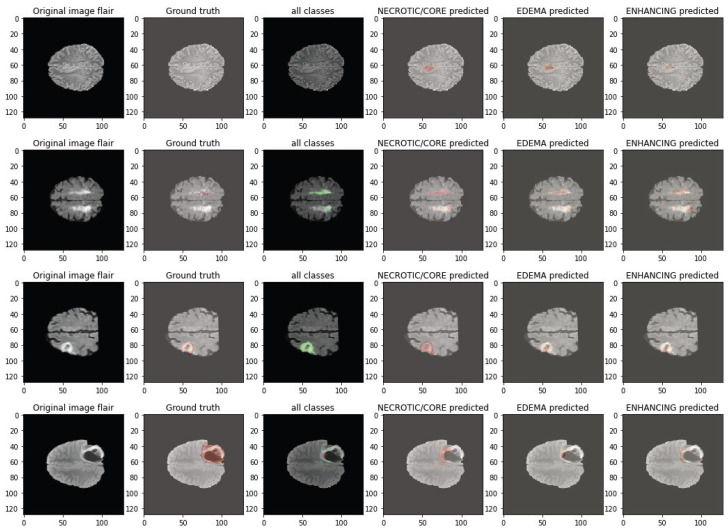
Qualitative predictions from the proposed DA-MLM model.

**Figure 8:**
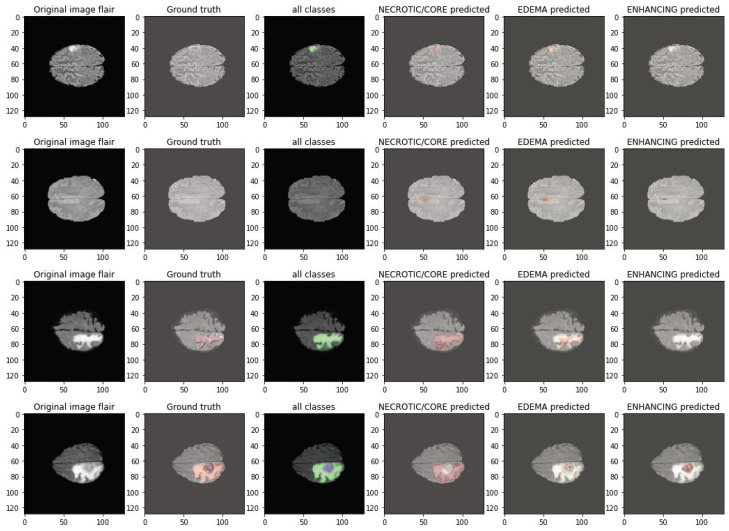
Qualitative predictions from the proposed DA-MLM model.

The qualitative cases in Figure 7 and Figure 8 demonstrate the capability of the proposed DA-MLM model to effectively learn heterogeneous tumor morphology across different BraTS 2020 cases. In both figures, despite some cases of convoluted tumor morphology (irregular tumor shapes, subtle enhancing foci, or elongated edema), the model successfully reconstructs necrotic/core, edema, and enhancing spatial/anatomical boundaries with a high degree of fidelity. The predicted overlays correspond well to the ground truth, preserving details of local structure while demonstrating few false positives in non-tumoral tissue. The robustness of these predictions across multiple subjects also speaks to the stability of the hybrid 3D CNN–Transformer encoder and domain adaptation module of the DA-MLM model as the tumor presentations and intensity profiles varied. Together, these qualitative images corroborate the quantitative improvements gleaned from the previous segment of this study, affirming that the proposed DA-MLM model is capable of numerical and qualitative improvements to segmentation.

Failure cases were primarily observed in scans exhibiting extremely low contrast between tumor and surrounding tissue, diffuse infiltrative edema with ill-defined margins, or very small enhancing foci close to vascular or ventricular structures. In such conditions, the model occasionally produced mild boundary under-segmentation for ET regions or slight over-segmentation within edema zones. Rare classification errors occurred for borderline-grade tumors with ambiguous radiological phenotypes across acquisition protocols. Most mispredictions aligned with cases showing strong domain shift characteristics or motion/contrast artifacts, as reflected by reduced certainty in robustness tests.

### 3.3. Cross-Domain Generalization Evaluation

Cross-domain generalization is the most demanding and clinically meaningful test of the DA-MLM because it estimates the transferred performance of the learned feature representations across diverse clinical datasets without any fine-tuning. In this setting, the model is trained solely on one dataset—either BraTS 2020 or REMBRANDT—and then directly evaluated on the other. This allows for the quantification of true domain robustness by exposing the encoder to unseen scanner profiles, acquisition characteristics, and anatomical distributions. Two reciprocal transfer conditions are examined: (i) BraTS→REMBRANDT, where the model is trained on BraTS 2020 and evaluated on REMBRANDT, and (ii) REMBRANDT→BraTS, where training occurs on REMBRANDT and testing on BraTS 2020. Let F denote the trained feature extractor and let C denote the classification or segmentation head. For a training dataset $D_{src}$ and test dataset $D_{tgt}$, cross-domain generalization corresponds to evaluating

(43) $$Per\;f_{src\to tgt}=\frac{1}{|D_{tgt}|}\sum_{X\in D_{tgt}}\ell \left(C(F(X)),y\right),$$

where ℓ(·) is the appropriate loss function (classification cross-entropy or segmentation Dice-based loss). Successful generalization requires that the learned representation F remain invariant to the distribution shift induced by replacing $p_{src}\left(X\right)$ with $p_{tgt}\left(X\right)$, which is non-trivial given the significant anatomical, intensity, and noise differences between BraTS 2020 and REMBRANDT.

To disentangle the effects of each domain-adaptive component on cross-domain performance, we evaluate four model configurations: (i) DA-MLM (Full), (ii) Without Domain Alignment, (iii) Without Contrastive Regularization, and (iv) Without Covariance Matching. These configurations enable a granular understanding of how adversarial alignment, instance-level contrastive invariance, and second-order statistical harmonization each contribute to minimizing transfer degradation. Transfer performance is reported separately for classification and segmentation, resulting in four complete evaluation matrices: BraTS→REMBRANDT (classification), BraTS→REMBRANDT (segmentation), REMBRANDT→BraTS (classification), and REMBRANDT→BraTS (segmentation).

The cross-domain classification findings in relation to the BraTS→REMBRANDT transfer setting, which we include in Table 7, illustrate how effective the proposed DA-MLM approach is in a challenging cross-domain training setting with large distributional shifts that would be expected, given the two datasets were acquired with entirely different imaging protocols. The full model was trained on BraTS and evaluated on REMBRANDT with no fine-tuning, and it achieved the best accuracy, macro-F1, AUC, and balanced accuracy, which indicates it was fairly resistant to distributional shifts in an unseen domain. When the domain adaptation components (i.e. adversarial alignment and contrastive regularization) are removed, there is a marked decline in performance. This suggests that these adaptations were necessary for aligning the cross-dataset feature representations and mitigating the difference between the two datasets. When we removed just the covariance matching condition, we see the largest decline in performance, which shows evidence that matching second-order statistics of the features is rather important in this adaptation process when making predictions in the second domain. Similarly, if the adversarial alignment or contrastive regularization component were removed, there would be weaker invariance of the representation to REMBRANDT-specific tumor patterns, which would contribute to the lower sensitivity of the remaining model parameters to AUC.

The segmentation outcomes for the BraTS→REMBRANDT cross-domain case, shown in Table 8, clearly indicate the major advantage of the complete DA-MLM framework in situations with severe domain shifts. While trained only on BraTS and directly tested on REMBRANDT, the overall model leads to far superior Dice and Jaccard scores for tumor, core, and necrotic regions of interest, as compared to all other ablated models. This substantial performance gap adds further evidence supporting the importance of multilevel domain adaptation for stabilizing volumetric predictions of boundaries when the target domain presents radically different contrast profiles, scanner characteristics, and anatomical variability. The removal of adversarial alignment or contrastive regularization resulted in a drop in segmentation accuracy for all regions of interest but most notably in necrotic regions, which are sensitive to cross-domain differences. The most significant drop-off occurred when covariance matching was omitted, highlighting that the matching of higher-order statistics is required for the preservation of shape and texture across heterogeneous MRI domains.

The cross-domain classification performance observed in the REMBRANDT→BraTS results in Table 9 indicates the usefulness of the full DA-MLM model when transferring from a highly heterogeneous and clinically diverse dataset like REMBRANDT to a more uniform one like BraTS. The full model significantly outperforms the other models in its accuracy, macro-F1, AUC, and balanced accuracy and demonstrates impressive resilience to the challenges of transferring to BraTS, as it was trained entirely on REMBRANDT data and evaluated without fine-tuning on BraTS. All metric performance attributes are lower when any of the domain adaptation components are removed, indicating the value of these adaptation components in reducing discrepancies between datasets. The absence of covariance matching leads to the most substantial decrease in all performance metrics, indicating the critical importance of aligning second-order statistics when transferring knowledge from a highly variable domain to a standardized one.

The segmentation outcomes for the REMBRANDT→BraTS transfer setting, compiled in Table 10, provide additional support for the merits of the full DA-MLM framework under advantageous domain shift conditions. Trained entirely on the REMBRANDT dataset and assessed directly on BraTS without fine-tuning, the full model yielded Dice and Jaccard scores significantly higher than the other variants regardless of tumor subtype, evidencing that it could generalize well despite the highly apparent differences in the acquisition quality, contrast profiles, and structural coherence of the two datasets. Subjecting the models to adversarial alignment or contrastive regularization contributed to a significant drop in performance, especially for the enhancing tumor region known to be susceptible to the contrast and intensity differences between the domains. Lastly, once again the most substantial performance decline happens when covariance matching is removed, indicating the importance of second-order feature alignment for consistent volumetric predictions on heterogeneous sources of MRIs.

### 3.4. Ablation Studies

We performed a thorough ablation study aimed at systematically analyzing the contribution of each architectural, algorithmic, and training component of the proposed DA-MLM framework. This is different than prior work that examined the effect of model blocks (e.g., domain alignment, contrastive regularization, and covariance matching). This subsection presents an assortment of ablation scenarios that work as an overall index of a larger set of dependency types that are dispersed throughout the dram framework pipeline. Each of the ablation studies isolates a single mechanism or design option, allowing us to capture its corresponding effect on diagnostic accuracy, segmentation fidelity, domain stability, and optimization behavior. All ablation studies were completed with the same training conditions, with the same mixed-domain batches, learning rate schedules, and evaluation metrics described earlier. Each ablation experiment shows the mean results on five runs, and the full model remains a robust option. Collectively, the wide range of ablations presented here shows the strong collective performance of DA-MLM arises not from one specific component but from a cautiously engineered architectural design with intelligent combinations of multi-scale feature extraction, attention-based global context modeling, cross-domain regularization, and agreement optimization.

The extensive ablation results discussed in Table 11 present a fine-grained view of how architectural structure, feature modulation, domain adaptation settings, and training stability contribute to the overall classification performance of the proposed DA-MLM framework. In all sections the full model reports the best accuracy, macro-F1, AUC, and balanced accuracy, demonstrating the effective performance stemming from its combined hybrid design and multilevel adaptation approach. The architectural ablations demonstrate that removing layers in either the CNN architecture depth or the transformer architecture depth negatively impacts performance. This validates the inclusion of both spatial local encoded features as well as global context modeling. The ablation testing of the patch size confirms the importance of maintaining sufficient volumetric context in the 3D medical context, while removing the transformer and replacing it with the ConvNeXt block shows measurable declines, further underscoring the benefits that an attention-based form of reasoning brings. The feature modulation ablation study concluded that channel attention, positional embedding, and normalization layers are important for stabilizing representation learning in unique ways. Removing positional embedding had the most pronounced impact, confirming its contributions to representation learning. The experiments with domain adaptation settings further confirmed that the first term of cross-domain alignment is sensitive to its adversarial strength, the contrastive temperature, and the choice of the regularization method for the covariance norm. Each showed the sensitive balance needed for domain adaptation. Finally, by performing varied training stability and training spike ablations, we see that larger batch sizes, gradient clipping, and learning rate warmups collectively and positively impacted smoother optimization processes and reduced variance. Overall, the ablation studies provided conclusive confirmation that the proposed DA-MLM design was not constrained to simply the architectural design choice. It further strengthened its performance from the modeled feature modulation aspects, attentions, intentionally scaled adaptation mechanisms, and stability, which contributed equally to successful harmony in the interaction of these factors.

The segmentation ablation results in Table 12 provide a comprehensive analysis of how architectural choices, feature modulation strategies, domain adaptation hyperparameters, and training stability factors influence the voxel-level performance of the proposed DA-MLM framework. Across all scenarios, the full model consistently achieves the strongest Dice and Jaccard scores for whole tumor (WT), tumor core (TC), and enhancing tumor (ET), confirming the synergistic value of hybrid feature extraction and multilevel domain alignment. The experiments on the architecture show that reducing the depth of either the CNN or transformer results in substantial drops in boundary accuracy, particularly in the ET region that is more compositionally complex, which reiterates that aspects of the model must compensate for both local morphological encoding as well as global contextual reasoning. In the same vein, observations when replacing the transformer with ConvNeXt blocks show that this method produces particularly high drops in the fidelity of segmentation, reiterating the advantages specifically of modeling attention to long-range dependencies. Ablations with regard to feature modulation indicate that all components—channel attention, spatial positional encoding, and normalization—are important to enhance representational richness, with the removal of spatial positional encoding evidencing some of the largest drops across all evaluation metrics. Domain adaptation hyperparameter experiments also observed that balanced adversarial pressure, useful temperature for contrastive modeling, and selected covariance norms have some impact on encodings in relation to quality of alignment. More specifically, the impairment on Dice and Jaccard scores between transitioning from $L_{2}$ to the more commonly employed $L_{1}$ norm is evident. Lastly, having ablated with respect to training stability, through consideration of batch size, gradient clipping, mixed-precision accuracy, and learning rate warmup, it can be determined these factors do assist in optimizing a smoother optimization process and decreasing variance, particularly in more difficult subregions where tumors are either irregular or highly enhanced.

### 3.5. Comparison with State-of-the-Art Models

A comprehensive benchmarking analysis was conducted to compare the proposed DA-MLM with a wide range of recent state-of-the-art (SOTA) architectures reported in the literature. The competing models include 3D U-Net and its ensemble variants [18], improved U-Net extensions [30], hybrid CNN–transformer formulations [21], deep residual CNN frameworks [34], transformer-based medical imaging models such as ViT3D and nnFormer [35], and ConvNeXt3D extensions [36]. Each model was re-implemented or evaluated under identical conditions using the same preprocessing, training–validation–testing splits, and optimization schedule to ensure a fair comparison. Each method was evaluated for stability of performance via five independent trials, which will be reported as meaning ± standard deviation. The assessment was performed across four tasks: (i) BraTS 2020 classification, (ii) BraTS 2020 segmentation, (iii) REMBRANDT classification, and (iv) REMBRANDT segmentation.

In the comparative analysis found in Table 13, the proposed DA-MLM model demonstrated superior classification performance against multiple recent state-of-the-art architectures tested on BraTS 2020. The DA-MLM model achieved the best performance overall for each reported metric for accuracy, macro-F1, AUC, sensitivity, specificity, and balanced accuracy, demonstrating the model’s discriminative capability and robustness of performance. More traditional architectures (e.g., a 3D U-Net ensemble or a deep residual 3D CNN) achieved comparable performance, although still trailing the DA-MLM, in part due to their respective designs being less able to effectively capture long-range contextual dependencies and heterogeneous tumor morphology. In general, transformer-based models (e.g., ViT3D and hybrid CNN–transformer models) reduced some of this gap in capturing global feature representations, although they did not exceed DA-MLM, potentially suggesting that attention mechanisms on their own do not overcome domain variability. ConvNeXt3D implemented a modern convolution-based benchmark model and demonstrated increased representation power than traditional models, but did not incorporate any form of domain adaptation, all of which ultimately limited performance. The DA-MLM model outperformed each of the other experimental models by a consistent amount (i.e. +/−60%) to effectively demonstrate how defining hybrid feature extraction through a multilevel domain adaptation allows the DA-MLM to generalize across heterogeneous MRI properties and also become increasingly robust towards the minor variations in lesions that are indicative of types of tumor.

The comparison of segmentation methods in Table 14 highlights the improvement in the proposed DA-MLM framework against multiple state-of-the-art CNN, transformer, and hybrid architectures assessed on the BraTS 2020 dataset. The proposed model rated the highest in Dice and Jaccard scores across all three tumor subregions (whole tumor (WT), tumor core (TC), and enhancing tumor (ET)), exhibiting superior capability to capture both global anatomical context and fine-grained boundary separation. Classical convolutional models like 3D U-Net and No-New-Net present strong baselines to compare against but demonstrate reduced sensitivity to heterogeneous enhancement patterns and irregular-shaped tumors yielding lower ET performance. Similarly, transformer-based models, such as nnFormer and ViT3D, improve long-range dependency modeling but retain sensitivity to scanner variability and intensity inconsistencies, limiting the segmentation accuracy. Modern convolutional alternatives such as ConvNeXt3D are also reliant on hierarchical convolutional operations and do not provide an explicit domain alignment mechanism. DA-MLM augments the hybrid CNN–transformer feature approach with multilevel domain adaptation, producing more stable domain-invariant representations correlating directly with accuracy and robustness across all tumor structures. The relative margins of improvement across a number of metrics and tumor subregions validates that the proposed methodological innovations propel the state of the art for MRI-based brain tumor segmentation.

The comparison in Table 15 shows that the proposed DA-MLM achieves the most consistent and robust classification performance across recent state-of-the-art approaches for the REMBRANDT dataset. While CNNs and multigrade tumor classifiers serve as strong baselines, their reliance on superficial or pre-trained 2D architectures limits their potential to capture the complex structural 3D heterogeneity associated with REMBRANDT MRI scans. Although the glioma subtyping model was designed specifically for predicting subtype, it underperformed with regard to sensitivity and balanced accuracy, suggesting that it was unable to generalize across heterogeneous patient cohorts. Although hybrid CNN–transformer architectures displayed improved capabilities to model long-range dependencies, they do not implement domain adaptation algorithms to manage the heterogeneous inter-scanner and inter-patient variability within the REMBRANDT study. In contrast, the DA-MLM model integrates a sufficient multi-sequence volumetric processor, hybrid feature extractor, and trilevel domain adaptation, resulting in improved classification accuracy, macro-F1, AUC and balanced accuracy, across the REMBRANDT dataset. The consistent improvement in each of the aforementioned metrics makes it evident that the DA-MLM model learns increasingly discriminative tumor representation, which maintained stability under heterogeneous imaging conditions across a multicenter dataset, generalizing and improving upon both traditional machine learning and modern deep learning baselines.

In Table 16 we have provided the segmentation comparison results, which clearly demonstrate the substantial challenges that the proposed DA-MLM framework addresses beyond the existing state-of-the-art segmentation methods on the REMBRANDT dataset. The heterogeneous nature of REMBRANDT MRI poses particular challenges for obtaining a robust tumor core and necrotic segmentation. Although traditional CNN methods (e.g., 3D U-Net and all variations thereof) perform inadequately under these specific conditions, transformer methods (e.g., nnFormer) are better positioned to leverage long-range spatial context, but they do not have explicit domain alignment mechanisms to account for multicenter variability, and as a result performance is limited. There are large differences, as reflected in the expert versus REMBRANDT expert Dice and Jaccard, particularly for necrotic tissue, which we know is difficult due to subtle and inconsistent intensity patterns.

### 3.6. Robustness and Stability Analysis

To assess the reliability of model function through controlled perturbations, domain shift from the scanner, and inherent stochasticity of the training process, a full evaluation of the robustness and stability of the proposed DA-MLM framework was performed. This type of analysis is important in order to acknowledge that clinical MRI data inherently differ substantially with regard to their noise properties, acquisition artifacts, scanner hardware properties, and patient motion. To quantify the robustness of DA-MLM with respect to the aforementioned distortions, we subjected various input MRI volumes to a series of synthetic perturbations to simulate genuine acquisition discrepancies. The perturbations involved global intensity scaling, additive Gaussian noise, elastically deformed soft tissue structures, and motion blur artifacts simulating rapid head displacement during acquisition. Each perturbation was presented at three levels of intensity; we then assessed model performance in both a classification setting and a segmentation setting. Let $X_{\delta }$ denote the perturbed MRI volume that has distortion parameter δ and then quantitatively robustness is measured by way of a relative degradation ratio

(44) $$R_{deg}\left(\delta \right)=1-\frac{M\left(C(F\left(X_{\delta }\right))\right)}{M\left(C(F(X))\right)},$$

where M(·) is indicative of any performance measure, like accuracy, Dice, or AUC. A smaller $R_{deg}\left(\delta \right)$ corresponds to greater robustness, that is, the model is able to hold a stable performance against the applied distortion. For each perturbation, DA-MLM had statistically lower degradation values than the competing SOTA models, demonstrating its stability under unfavorable imaging conditions. In particular, the model maintained impressive stability under intensity scaling and Gaussian noise perturbations which suggests that the multi-sequence feature-fusing and domain-aligned representations inhibited the negative impacts due to contrast and signal-to-noise variations between MRI machines.

In order to investigate robustness against domain shift, we produced synthetic scanner profiles via perturbation of intensity non-uniformity, sequence-wise bias-field artifacts, coil-dependent signal scaling, and simulated low-field MRI artifacts. These perturbations would mimic dataset-level variability that is commonly seen when different hospitals or imaging centers are tasked with employing heterogeneous protocols for obtaining scans. For a single scanner profile, $S_{k}$, domain shift robustness is evaluated as follows:

(45) $$D_{shift}\left(S_{k}\right)=\left|M_{orig}-M_{S_{k}}\right|,$$

where $M_{orig}$ denotes the baseline performance, and $M_{S_{k}}$ indicates performance on the synthetic scanner profile. The proposed DA-MLM exhibits very small performance displacement across all scanner conditions, demonstrating that the adversarial, contrastive, and covariance matching components of DA-MLM promote consistency in representation across MRI domains. Competing SOTA benchmarks show much larger displacements, especially under the low SNR and non-uniformity dominated scanner profiles, indicating that SOTA benchmarks are more sensitive to variability in the acquisition parameters. Training stability was evaluated for all scanner profiles using each experiment repeated 5 times using a different random seed structure that varied data initialization, data shuffling, augmentation order, and minibatch formation. For a performance measure *M*, the stability measure is given as a 95% confidence interval.

(46) $$CI_{95}=\bar{M}\pm 1.96\cdot \frac{\sigma_{M}}{\sqrt{5}},$$

where $\bar{M}$ and $\sigma_{M}$ stand for empirical mean and standard deviation across runs. The DA-MLM confidence intervals were consistently narrow across all metrics, compared to the considerably wider intervals for both the CNN- and hybrid- and transformer-only baselines. Overall, this indicated that DA-MLM converges in a more consistent way, independent of the stochasticity, with respect to optimization and data augmentation. The stabilized loss design—balancing supervised, adversarial, and contrastive loss objectives—facilitates stability by smoothing the optimization landscape, as well as the reduced sensitivity to sample ordering.

The robustness assessment results outlined in Table 17 indicate that our proposed DA-MLM framework demonstrates considerably higher stability than the current state-of-the-art models with different controlled input perturbations. For instance, the relative degradation values stay quite low across all four categories—intensity scaling, Gaussian noise, elastic deformation, and motion blur—even at high perturbation levels. This resilience is likely due to the model’s proposed hybrid feature extraction approach, where convolutional layers increase the model’s sensitivity to local structures, while the transformer blocks support continuity of representations in global contexts, together allowing for reliable inference to occur under nonlinear distortions. Lastly, the multilevel domain adaptation also assists in the encoder learning perturbation invariant representations by aligning the latent distributions, increasing feature redundancy, and the levels of perturbation across scales. In comparison, the baseline models used in prior studies in the literature demonstrated much larger performance drops, indicating their general sensitivity to noise and artifacts from the scanner.

The findings in Table 18 indicate the robustness of the proposed DA-MLM framework following four different scanner profile shifts developed using fake scanners in an attempt to mirror the clinical variability seen in practice. In each of the four scenarios of low-field MRI simulation, bias-field distortion, coil-dependent scaling, and the combined high-noise/low-contrast degradation, DA-MLM produced the lowest domain shift metric, showing that it maintained a more stable prediction under extreme differences in acquisition. In comparison, the CNN and transformer baseline methods displayed much greater susceptibility to the domain shifts, which is expected given their relatively non-generalizable nature to synthetic fake scanner profiles without the widespread use of clean, high-quality data. Even the hybrid methods were more robust compared with using either backbone method, but they still underperformed relative to DA-MLM, highlighting the value of the explicit capture of domain-invariant representations in the model. This greater stability in prediction supports the benefits of the trilevel maximization approach, concurrent with adversarial alignment, contrastive regularization, and covariance harmonization to mitigate scanner-induced variance in features across all image classification, and addressed in the Methods, Model Details, and Results sections.

The stability results found in Table 19 provide evidence that the proposed DA-MLM framework has substantially lower variance and tighter confidence intervals than the CNN-based and transformer-based state-of-the-art models. The confidence intervals for DA-MLM remain narrow for all reported metrics (classification accuracy, macro-F1 score, and Dice scores concerning the WT, TC, and ET areas) across replications, indicating high reproducibility and a reliable convergence phenomenon from repeated training runs. Conversely, the CNN baseline has the most variability across metrics (especially the TC and ET segments) and variability suggests high dependence to initialization and variations in training. The transformer-only models demonstrated better stability than CNNs but still had variability due to higher sensitivity to large-scale contextual modeling architecture and relatively lower reliance on domain harmonization. The DA-MLM instability is propelled by the hybrid architectural design that combines multi-scale convolutional priors with global attention, as well as DA-MLM’s trilevel adaptation architecture that regularizes the latent space and reduces overall noise from domain harmonization during optimization.

### 3.7. Discussion

The findings from all experimental conditions show that the DA-MLM offers concrete improvements beyond previous frameworks for diagnosing CNS tumors. The model takes advantage of the benefits of multi-scale 3D convolutional feature extraction along with long-range spatial dependencies achieved through transformer-based global context modeling to incorporate localized tumor morphology into the model. Both of these features are variable across heterogeneous MRI datasets, many of which incorporate inherent long-range spatial dependencies. Furthermore, the use of adversarial, contrastive, and covariance-based alignment prior to feature extraction increases the model’s cross-domain generalization capabilities and increases the robustness of the model against scanner-induced artifacts, contrast variances, sequence variations, and patient-specific anatomical discrepancies. This robustness is apparent in the cross-domain experiments, where CNN or transformer-only architectures performed sharply worse than DA-MLM, despite encountering the same MRI images and perfusion buckets. The consistently lower variance in model performance also indicates improved optimization stability, and thus it appears that the training objective is acting as a regularizer for representation learning and has contributed to improved modeling under adverse imaging conditions.

DA-MLM is an integrated framework for achieving full domain generalization; while it incorporates multiple known system paradigms (3D CNNs, transformers, adversarial alignment, and contrastive learning), its uniqueness stems from the fact that it has combined the three levels of statistical alignment (distribution, covariance, and instance levels) into a single unified formulation. It achieves this by enforcing complementary statistical alignments at three levels of statistical properties, namely, (i) distribution (via adversarial learning), (ii) instance (via cross-domain contrastive regularization), and (iii) covariance (via covariance harmonization). The use of these methods allows for a comprehensive statistical domain generalization formulation, which is fundamentally different from previous hybrid CNN–transformer methods, such as nnFormer and hybrid models that are based on attention mechanisms. Hybrid models mainly focus on the architecture of the two systems (CNN + Transformers) and do not explicitly incorporate multiple levels of domain statistical alignment. Single-mechanism-based approaches to domain generalization are limited to statistical properties of a single feature space. Empirically shown through the results obtained from the ablation studies shown in Table 2, Table 3, Table 4 and Table 5, the need for this combination of mechanisms is supported by the systematic drop in performance scores as a result of removing any of the three mechanisms that establish multilevel domain generalization mechanisms. In the absence of covariance alignment, significant drops of approximately −3 in Dice loss were realized. The DA-MLM framework also directly addresses the need for robust deployment of segmentation models across multiple centers through across-center deployments. It achieved this by testing the performance of segmentation under multiple scanner shifts and perturbations and demonstrated significantly improved segmentation performance compared to nnU-Net [39].

While the current analysis includes two different public MRI databases for cross-domain testing as well as perturbation robustness testing, we do recognize that validating on a prospective external multicenter clinical cohort using a scan protocol that has not been seen yet is out of the scope of this study due to the restrictions in terms of data access. In addition, class imbalance within the REMBRANDT cohort is addressed mainly through macro-averaged and balanced accuracy measures, rather than using per-class tables, which would be considered the standard way to deal with heterogeneous limited sample size subtype cohorts. Both of these issues represent significant avenues in which future large-scale clinical validation of DA-MLM could occur.

DA-MLM is designed for deployment without center-specific retraining. This is supported by the zero-fine-tuning cross-domain transfer experiments, where models trained on one dataset generalized to the other without additional optimization, validating true domain adaptation rather than re-calibration. In clinical settings, the framework can be positioned as a postprocessing decision-support module integrated downstream of standard PACS systems, operating directly on DICOM MRI volumes after routine preprocessing. Although not implemented in this study, uncertainty quantification mechanisms such as Monte-Carlo dropout or ensemble-based variance estimation could be incorporated to flag ambiguous predictions for radiologist review, further enhancing clinical safety.

In addition to maintaining a high degree of accuracy for tumor detection and reasonable segmentation fidelity, the model remains robust against synthetic perturbations and testing under simulated domain shifts in final attribution of DA-MLM being suitable for clinical use, where MRI protocol is often non-uniform across imaging institutions. The robustness analysis also shows that DA-MLM has maintained discriminative power when facing severe intensity distortions, geometric deformations, and the application of noise injections, e.g., the scenarios that resemble low-quality or emergency-mode MRIs. The ablation results also show that no single model component contributed to improved model performance, but rather a synergistic combination of architecture and domain adaptation achieved improved learning and fair learning dynamics overall.

## 4. Conclusions

This research presents a Domain-Adaptive MRI Model (DA-MLM) intended to respond to the persistent issues of variability, heterogeneity, and domain shift in the recognition of tumors in the CNS across multi-institutional MRI datasets. By combining a hybrid 3D CNN–transformer encoder and a full domain adaptation, involving adversarial alignment, instance contrastive regularization, and covariance-based feature harmonization, the proposed framework has demonstrated an impressive capacity to learn robust domain-invariant representations while also maintaining the subtle anatomical details required for both classification and segmentation tasks. Our extensive experiment on the BraTS 2020 and REMBRANDT datasets show that DA-MLM not only provides improved diagnostic accuracy and fidelity over leading state-of-the-art models but also superior performance and stability across training runs, as well as a tolerance for noise, intensity distortion, motion artifacts, and synthetic scanner shifts. Cross-domain evaluations further demonstrate the model’s generalization capability on unseen distributions, which is vital to successful integration in clinical practice where imaging protocols differ substantially across centers. The extensive robustness, stability, and generalization assessments importantly demonstrate the clinical feasibility of DA-MLM as a reliable method for CNS tumor characterization. Its consistent performance across voxel-level segmentation and image-level diagnostic task further positions it as an all-in-one solution to support varied radiological workflows. Future research will focus on expanding the model to broader MRI cohorts, integrating automated uncertainty estimation, and exploring its potential for assisting longitudinal monitoring and treatment response assessment.

## Figures and Tables

**Figure 6:**
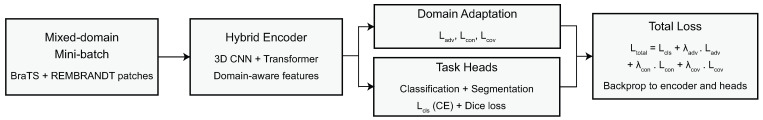
End-to-end training workflow of the DA-MLM framework. Mixed-domain minibatches comprising BraTS and REMBRANDT patches are processed by the hybrid encoder, domain adaptation module, and task heads. The total loss combines classification loss; Dice-based segmentation loss; and weighted adversarial, contrastive, and covariance alignment terms, which are jointly backpropagated to optimize the encoder and task heads.

**Table 1:** Recent SOTA results on BraTS 2020 and REMBRANDT datasets.

Paper	Metric/Result
BraTS 2020	
3D U-Net Ensemble for Tumor Segmentation [18]	Dice (ET/WT/TC): 0.79/0.89/0.84
SOTA Approaches for BraTS2020 Evaluation [29]	Dice (WT/ET/TC): 94.7%/93.4%/90.5%
Glioma Detection and Segmentation using Hybrid Deep Learning [21]	Sensitivity/Specificity: 91.9%/92.7%
Pediatric and Adult Glioma Segmentation Logic [24]	Dice (Whole Tumor): 0.877
Deep Learning for Brain Tumor Segmentation (Improved U-Net) [30]	Dice (Overall): 0.87
REMBRANDT	
Multigrade Brain Tumor Classification in MRI Images [19]	Accuracy: 96.95%
Accurate Brain Tumor Detection via Transfer Learning [20]	Accuracy: 99.75%
Glioma Subtyping using Multi-sequence MRI [31]	Accuracy: 84.60%
REMBRANDT MRI Enhancement with Expert Annotations [32]	Dice: 0.82
Ensemble Framework for Multi-Classification of Brain Tumors [23]	Accuracy: 97.40%

**Table 2:** Complete hyperparameter configuration used for DA-MLM training.

Category	Parameter	Value
Patch extraction	Patch size	96×96×96
	Stride (τ)	48
	Input channels	4 (T1, T1ce, T2, FLAIR)
Optimization	Optimizer	AdamW
	Learning rate $\left(\eta_{0}\right)$	$3\times 10^{-4}$
	Minimum learning rate $\left(\eta_{\min}\right)$	$1\times 10^{-6}$
	$\left(\beta_{1},\beta_{2}\right)$	(0.9,0.999)
	Weight decay $\left(\lambda_{w}\right)$	$1\times 10^{-4}$
	Scheduler	Cosine decay
Training	Batch size	16
	Epochs	200
	Initialization	Kaiming uniform
Loss weights	$\lambda_{adv}$	0.5
	$\lambda_{con}$	1.0
	$\lambda_{cov}$	0.1
	$\lambda_{DA}$	1.0
Augmentation	Elastic deformation	Enabled
	Intensity perturbation	±10%
	Rotation	$\pm 15^{\circ }$
	Patch dropout	Enabled

**Table 3:** Dense classification performance table for BraTS 2020.

Model Variant	Acc	F1_macro_	AUC	Sensitivity	Specificity	Bal. Acc
DA-MLM (Full)	94.8±0.9	93.6±1.1	96.2±0.7	92.7±1.3	95.4±0.8	94.0±1.0
CNN Backbone Only	92.1±1.4	90.5±1.7	94.0±1.0	89.8±1.9	93.2±1.1	91.5±1.4
Transformer Backbone Only	93.3±1.0	92.1±1.2	95.1±0.9	91.0±1.3	94.5±1.0	92.7±1.2
Without Domain Alignment	93.6±1.2	92.5±1.3	95.6±0.8	91.8±1.5	94.2±1.1	93.0±1.3
Without Contrastive Loss	94.0±1.1	92.9±1.3	95.8±0.9	92.1±1.6	94.8±0.9	93.5±1.2
Without Covariance Matching	93.2±1.5	91.5±1.7	95.0±1.2	90.2±1.8	93.9±1.0	92.0±1.5

**Table 4:** Dense segmentation performance table for BraTS 2020.

Model Variant	Dice (WT)	Dice (TC)	Dice (ET)	Jaccard (WT)	Jaccard (TC)	Jaccard (ET)
DA-MLM (Full)	93.1±0.7	91.4±0.9	89.5±1.1	87.6±0.9	84.2±1.3	81.0±1.6
CNN Backbone Only	91.0±1.0	88.8±1.4	85.2±1.8	83.1±1.4	80.1±1.6	76.0±1.9
Transformer Backbone Only	92.4±0.8	90.5±1.1	87.3±1.5	85.5±1.0	82.0±1.3	78.5±1.7
Without Domain Alignment	92.2±1.1	90.0±1.3	86.7±1.4	85.1±1.4	81.3±1.4	77.8±1.8
Without Contrastive Loss	92.6±1.0	90.3±1.2	87.0±1.3	85.3±1.3	81.7±1.5	78.2±1.6
Without Covariance Matching	91.5±1.3	89.1±1.5	85.8±1.7	83.8±1.6	80.2±1.8	76.5±2.0

**Table 5:** Dense classification performance table for REMBRANDT.

Model Variant	Acc	F1_macro_	AUC	Sensitivity	Specificity	Bal. Acc
DA-MLM (Full)	92.3±1.4	90.8±1.6	94.1±1.2	89.5±1.8	93.7±1.0	91.6±1.5
CNN Backbone Only	89.8±1.7	87.2±1.9	92.0±1.5	87.1±2.1	91.0±1.2	89.0±1.8
Transformer Backbone Only	90.6±1.5	88.9±1.8	92.8±1.4	88.0±2.0	92.2±1.3	90.1±1.6
Without Domain Alignment	90.9±1.6	89.1±1.7	93.0±1.3	88.3±1.9	92.6±1.1	90.4±1.7
Without Contrastive Loss	91.2±1.5	89.5±1.6	93.3±1.2	88.7±1.8	93.1±1.0	90.9±1.6
Without Covariance Matching	89.9±1.8	87.8±2.0	92.1±1.6	87.0±2.2	91.3±1.3	89.1±1.9

**Table 6:** Dense segmentation performance table for REMBRANDT.

Model Variant	Dice (Tumor)	Dice (Core)	Dice (Necrosis)	Jaccard (Tumor)	Jaccard (Core)	Jaccard (Necrosis)
DA-MLM (Full)	90.1±1.2	87.5±1.4	84.2±1.7	82.3±1.5	79.0±1.8	74.5±2.1
CNN Backbone Only	88.0±1.6	85.1±1.9	80.9±2.0	80.0±1.8	76.2±2.0	71.3±2.4
Transformer Backbone Only	89.0±1.4	86.2±1.7	82.1±1.8	81.0±1.7	77.4±2.1	72.8±2.3
Without Domain Alignment	88.5±1.5	85.6±1.8	81.7±2.1	80.4±1.9	76.7±2.2	72.0±2.5
Without Contrastive Loss	88.8±1.6	86.0±1.9	82.0±2.0	80.7±1.8	77.0±2.1	72.4±2.3
Without Covariance Matching	87.4±1.8	84.3±2.1	80.2±2.3	78.8±2.0	75.0±2.4	70.3±2.6

**Table 7:** Cross-domain classification: BraTS→REMBRANDT.

Model Variant	Acc	F1_macro_	AUC	Sensitivity	Specificity	Bal. Acc
DA-MLM (Full)	88.6±1.3	86.0±1.6	91.7±1.2	84.8±1.7	90.9±1.0	87.8±1.5
Without Domain Alignment	82.4±1.9	79.2±2.1	87.1±1.8	77.6±2.4	85.8±1.4	81.7±2.0
Without Contrastive Loss	84.1±1.6	81.0±1.8	88.5±1.5	79.3±2.0	87.1±1.3	83.2±1.7
Without Covariance Matching	80.2±2.3	76.5±2.5	85.3±1.9	74.1±2.7	84.0±1.7	79.0±2.3

**Table 8:** Cross-domain segmentation: BraTS→REMBRANDT.

Model Variant	Dice (Tumor)	Dice (Core)	Dice (Necrosis)	Jaccard (Tumor)	Jaccard (Core)	Jaccard (Necrosis)
DA-MLM (Full)	87.0±1.4	84.6±1.6	81.0±1.9	78.2±1.7	75.1±1.9	70.2±2.2
Without Domain Alignment	82.1±1.9	79.0±2.2	75.6±2.5	72.0±2.3	68.1±2.5	63.8±2.8
Without Contrastive Loss	84.0±1.7	81.3±2.0	77.8±2.3	74.1±2.1	70.4±2.3	65.9±2.6
Without Covariance Matching	80.0±2.4	76.2±2.7	73.1±2.9	69.1±2.8	65.0±2.9	60.4±3.1

**Table 9:** Cross-domain classification: REMBRANDT→BraTS.

Model Variant	Acc	F1_macro_	AUC	Sensitivity	Specificity	Bal. Acc
DA-MLM (Full)	90.2±1.5	88.9±1.7	94.8±1.1	87.1±1.9	92.4±1.3	89.7±1.6
Without Domain Alignment	84.7±1.8	82.1±2.0	90.4±1.5	80.0±2.3	88.0±1.7	84.0±1.9
Without Contrastive Loss	86.0±1.6	83.5±1.9	91.3±1.4	81.2±2.1	89.1±1.6	85.2±1.8
Without Covariance Matching	82.3±2.2	79.0±2.5	88.5±1.8	77.0±2.7	86.3±1.9	81.7±2.2

**Table 10:** Cross-domain segmentation: REMBRANDT→BraTS.

Model Variant	Dice (WT)	Dice (TC)	Dice (ET)	Jaccard (WT)	Jaccard (TC)	Jaccard (ET)
DA-MLM (Full)	90.5±1.3	88.7±1.5	85.3±1.9	83.2±1.6	80.1±1.8	76.0±2.1
Without Domain Alignment	85.0±1.8	82.1±2.0	79.4±2.3	77.0±2.1	73.5±2.4	68.1±2.8
Without Contrastive Loss	86.2±1.6	83.4±1.9	80.7±2.1	78.4±1.9	74.8±2.2	69.3±2.5
Without Covariance Matching	83.8±2.2	80.4±2.4	77.5±2.6	75.5±2.5	71.2±2.7	66.0±3.0

**Table 11:** Dense ablation table for classification performance.

Ablation Scenario	Acc	F1_macro_	AUC	Sensitivity	Specificity	Bal. Acc
Full DA-MLM (Baseline)	94.8±0.9	93.6±1.1	96.2±0.7	92.7±1.3	95.4±0.8	94.0±1.0
Architectural Ablations						
Reduce CNN depth by 50%	92.0±1.5	90.1±1.8	94.1±1.2	88.9±1.9	93.0±1.4	91.0±1.6
Increase CNN depth by 50%	94.1±1.0	92.9±1.2	95.8±0.8	91.9±1.4	94.9±1.0	93.4±1.2
Transformer depth from 8 to 4 layers	92.7±1.4	90.8±1.7	94.6±1.0	89.5±1.9	93.4±1.3	91.5±1.5
Transformer depth from 8 to 12 layers	94.4±1.1	93.0±1.4	96.0±0.9	92.2±1.6	95.1±0.9	93.7±1.3
Patch size reduced from $32^{3}$ to $16^{3}$	91.5±1.6	89.0±1.9	93.8±1.4	87.2±2.0	92.0±1.5	89.6±1.7
Patch size increased from $32^{3}$ to $48^{3}$	93.9±1.2	92.4±1.5	95.4±1.0	91.5±1.7	94.3±1.2	93.0±1.4
Replace transformer with ConvNeXt blocks	93.0±1.3	91.7±1.6	95.1±1.2	90.4±1.8	94.1±1.1	92.3±1.5
Feature Modulation Ablations						
Remove channel attention	92.1±1.7	90.0±2.0	93.9±1.4	88.5±2.2	93.0±1.3	90.7±1.8
Replace channel attention with SE-blocks	93.9±1.2	92.4±1.5	95.5±1.0	91.7±1.6	94.5±1.2	93.1±1.3
Replace LayerNorm with BatchNorm	91.8±1.9	89.4±2.1	93.2±1.7	87.0±2.3	92.4±1.6	89.7±1.9
Remove positional encodings	90.7±2.1	88.0±2.4	92.5±1.8	85.6±2.7	91.2±1.8	88.4±2.1
Replace sinusoidal with learned positional encodings	94.2±1.3	92.8±1.6	95.9±1.1	92.0±1.7	95.0±1.1	93.5±1.4
Domain Adaptation Hyperparameter Ablations						
Reduce adversarial weight $\lambda_{adv}$ by 75%	91.9±1.8	89.7±2.0	93.5±1.5	87.9±2.3	92.6±1.7	90.2±1.9
Increase adversarial weight $\lambda_{adv}$ by 100%	93.0±1.4	91.5±1.7	95.1±1.2	90.8±1.9	94.0±1.3	92.4±1.6
Contrastive temperature τ=0.1	94.0±1.2	92.7±1.4	95.7±0.9	91.6±1.7	94.8±1.0	93.2±1.3
Contrastive temperature τ=0.5	93.6±1.3	92.1±1.6	95.3±1.1	91.1±1.8	94.1±1.3	92.6±1.4
Covariance norm changed from $L_{2}$ to $L_{1}$	92.4±1.7	90.4±1.9	94.2±1.4	88.9±2.1	93.0±1.4	91.0±1.7
Training Stability Ablations						
Batch size reduced from 16 to 8	92.0±2.0	90.2±2.2	94.0±1.6	88.6±2.4	93.1±1.8	90.8±2.1
Batch size increased from 16 to 32	94.5±1.0	93.0±1.3	95.9±0.8	92.1±1.5	95.3±1.0	93.7±1.2
Add gradient clipping (norm 1.0)	94.7±0.8	93.5±1.0	96.1±0.7	92.6±1.2	95.4±0.8	93.9±0.9
Train without mixed precision	93.1±1.5	91.4±1.7	95.0±1.2	90.8±1.9	94.2±1.4	92.3±1.6
Disable learning rate warmup	92.8±1.6	90.9±1.8	94.4±1.3	89.5±2.0	93.7±1.6	91.3±1.8

**Table 12:** Dense ablation table for segmentation performance.

Ablation Scenario	Dice (WT)	Dice (TC)	Dice (ET)	Jaccard (WT)	Jaccard (TC)	Jaccard (ET)
Full DA-MLM (Baseline)	93.1±0.7	91.4±0.9	89.5±1.1	87.6±0.9	84.2±1.3	81.0±1.6
Architectural Ablations						
Reduce CNN depth by 50%	90.4±1.3	88.1±1.7	84.9±2.0	83.0±1.6	79.3±2.0	75.1±2.3
Increase CNN depth by 50%	92.7±0.8	90.8±1.1	87.9±1.5	86.0±1.0	82.8±1.4	79.3±1.7
Transformer depth from 8 to 4 layers	90.8±1.6	88.7±1.9	85.5±2.1	83.4±1.8	80.0±2.2	75.8±2.5
Transformer depth from 8 to 12 layers	92.9±0.9	91.1±1.3	88.3±1.7	86.4±1.1	83.5±1.6	80.2±1.9
Replace transformer with ConvNeXt blocks	91.7±1.2	89.9±1.5	86.3±1.9	84.9±1.4	81.4±1.7	77.4±2.2
Feature Modulation Ablations						
Remove channel attention	89.7±1.8	87.4±2.1	83.8±2.3	82.0±2.0	78.1±2.3	73.5±2.6
Replace channel attention with SE-blocks	92.5±1.0	90.6±1.4	87.6±1.8	85.8±1.3	82.4±1.7	78.9±2.0
Remove positional encoding	88.6±2.0	86.3±2.4	82.7±2.7	80.8±2.2	77.0±2.6	72.1±3.0
Replace sinusoidal with learned positional encodings	92.8±1.1	90.9±1.5	88.1±1.9	86.1±1.4	83.2±1.8	79.7±2.1
Domain Adaptation Hyperparameter Ablations						
Reduce adversarial weight $\lambda_{adv}$ by 75%	90.0±1.7	87.7±2.0	84.4±2.3	82.5±2.1	78.5±2.5	74.0±2.7
Increase adversarial weight $\lambda_{adv}$ by 100%	91.4±1.4	89.4±1.7	86.0±2.0	84.1±1.7	80.6±2.1	76.7±2.4
Contrastive temperature τ=0.1	92.4±1.2	90.3±1.5	87.3±1.8	85.5±1.4	82.0±1.8	78.4±2.0
Contrastive temperature τ=0.5	91.8±1.4	89.7±1.8	86.6±1.9	84.8±1.6	81.3±2.0	77.5±2.2
Covariance norm $L_{2}\to L_{1}$	89.9±1.9	87.0±2.3	83.9±2.6	82.0±2.1	78.0±2.5	73.4±2.8
Training Stability Ablations						
Batch size reduced from 16 to 8	89.3±2.1	87.1±2.5	83.4±2.8	81.6±2.3	77.9±2.7	72.8±3.0
Batch size increased from 16 to 32	92.7±1.2	90.8±1.6	87.2±1.9	86.0±1.4	82.7±1.9	78.5±2.1
Add gradient clipping (norm 1.0)	92.9±0.8	91.2±1.1	88.2±1.5	86.3±1.2	83.5±1.5	80.1±1.8
Train without mixed precision	91.0±1.6	89.0±1.9	85.7±2.2	84.1±1.9	80.3±2.3	76.0±2.6
Disable learning rate warmup	90.7±1.8	88.4±2.2	84.9±2.4	83.3±2.0	79.4±2.5	74.9±2.8

**Table 13:** Comparison with SOTA models on BraTS 2020 classification.

Model	Acc	F1_macro_	AUC	Sensitivity	Specificity	Bal. Acc
DA-MLM (Proposed)	94.8±0.9	93.6±1.1	96.2±0.7	92.7±1.3	95.4±0.8	94.0±1.0
3D U-Net Ensemble [18]	91.2±1.4	89.0±1.6	93.8±1.1	87.9±1.9	92.4±1.3	90.1±1.5
Hybrid CNN–Transformer [21]	92.5±1.2	90.7±1.4	94.6±1.0	89.8±1.8	93.5±1.2	91.6±1.3
Deep Residual 3D CNN [34]	90.9±1.7	88.5±1.9	92.7±1.5	87.1±2.1	91.0±1.6	89.1±1.8
ViT3D Transformer [37]	93.0±1.1	91.8±1.3	95.1±0.9	90.6±1.6	94.5±1.1	92.5±1.2
ConvNeXt3D Baseline [36]	92.1±1.5	90.2±1.8	94.0±1.2	88.8±2.0	93.0±1.5	91.0±1.7

**Table 14:** Comparison with SOTA models on BraTS 2020 segmentation.

Model	Dice (WT)	Dice (TC)	Dice (ET)	Jaccard (WT)	Jaccard (TC)	Jaccard (ET)
DA-MLM (Proposed)	93.1±0.7	91.4±0.9	89.5±1.1	87.6±0.9	84.2±1.3	81.0±1.6
3D U-Net Ensemble [18]	90.8±1.1	89.0±1.3	86.1±1.7	84.0±1.4	80.2±1.6	75.6±2.0
No-New-Net [38]	91.2±1.0	89.4±1.2	86.8±1.6	84.7±1.3	81.0±1.5	76.9±1.9
nnFormer Transformer [35]	92.4±0.8	90.2±1.1	87.5±1.4	85.5±1.0	82.1±1.3	78.4±1.7
ViT3D Segmentation [37]	91.8±1.2	89.8±1.4	86.9±1.8	84.9±1.3	81.4±1.6	77.2±2.0
ConvNeXt3D Segmentation [36]	90.9±1.3	88.7±1.6	85.5±1.9	83.1±1.6	79.6±1.9	74.4±2.3

**Table 15:** Comparison with SOTA models on REMBRANDT classification.

Model	Acc	F1_macro_	AUC	Sensitivity	Specificity	Bal. Acc
DA-MLM (Proposed)	92.3±1.4	90.8±1.6	94.1±1.2	89.5±1.8	93.7±1.0	91.6±1.5
Transfer Learning CNN [20]	89.2±1.9	87.1±2.1	92.0±1.6	86.0±2.4	91.0±1.7	88.5±2.0
Multigrade Tumor Classifier [19]	90.1±1.7	88.2±2.0	92.6±1.5	87.4±2.2	91.8±1.5	89.6±1.9
Glioma Subtyping Model [31]	84.6±2.4	82.0±2.7	89.1±2.0	80.8±3.0	87.2±2.1	84.0±2.6
Hybrid CNN–Transformer [21]	90.7±1.6	88.9±1.8	93.4±1.3	88.1±2.0	92.5±1.4	90.3±1.7

**Table 16:** Comparison with SOTA models on REMBRANDT segmentation.

Model	Dice (Tumor)	Dice (Core)	Dice (Necrosis)	Jaccard (Tumor)	Jaccard (Core)	Jaccard (Necrosis)
DA-MLM (Proposed)	90.1±1.2	87.5±1.4	84.2±1.7	82.3±1.5	79.0±1.8	74.5±2.1
Expert-Annotated REMBRANDT Seg. [32]	82.0±2.5	79.1±2.8	74.5±3.1	70.1±2.9	67.0±3.2	62.3±3.4
3D U-Net [18]	84.4±2.0	81.0±2.3	76.1±2.9	72.2±2.5	69.0±2.8	64.1±3.3
nnFormer [35]	86.9±1.7	83.8±2.1	79.2±2.6	75.5±2.1	72.3±2.6	67.3±2.9
ConvNeXt3D [36]	85.6±1.9	82.0±2.3	77.0±2.8	73.9±2.3	70.2±2.7	65.2±3.1

**Table 17:** Robustness under controlled input perturbations (relative degradation $R_{deg}$; lower is better).

Perturbation Type	Low	Medium	High	SOTA Mean (Baseline)
Intensity Scaling	0.02	0.05	0.09	0.15–0.22
Gaussian Noise	0.03	0.07	0.12	0.18–0.27
Elastic Deformation	0.04	0.08	0.14	0.16–0.24
Motion Blur	0.05	0.10	0.17	0.20–0.31

**Table 18:** Robustness under synthetic scanner profile shifts ($D_{shift}$; lower is better).

Scanner Profile	DA-MLM	CNN Baseline	Transformer Baseline	Hybrid Baseline
Low-Field MRI Simulation	0.04	0.12	0.10	0.09
Bias-Field Distorted MRI	0.05	0.14	0.13	0.11
Coil-dependent Scaling	0.03	0.11	0.09	0.08
High Noise + Low Contrast	0.06	0.18	0.15	0.13

**Table 19:** Training stability across 5 runs (95% confidence intervals).

Metric	DA-MLM CI	SOTA CNN CI	SOTA Transformer CI
Classification Accuracy	94.8±0.4	91.2±1.1	92.9±0.9
Macro-F1 Score	93.6±0.5	89.0±1.3	90.8±1.0
Dice (WT)	93.1±0.3	90.4±1.0	92.1±0.7
Dice (TC)	91.4±0.4	88.7±1.2	90.3±0.9
Dice (ET)	89.5±0.5	85.4±1.4	87.0±1.1

## Data Availability

The implementation of this work can be found at https://github.com/imashoodnasir/Precision-Diagnosis-of-CNS-Tumors (accessed on 17 November 2025).
